# Poly(allylamine)/tripolyphosphate nanocomplex coacervate as a NLRP3-dependent systemic and mucosal adjuvant for vaccines

**DOI:** 10.3389/fimmu.2026.1751634

**Published:** 2026-04-17

**Authors:** Gastón P. Rizzo, Rodrigo C. Sanches, Camila Chavero, Daiana S. Bianchi, Eugenia Apuzzo, Santiago E. Herrera, Maximiliano L. Agazzi, M. Lorena Cortez, Waldemar A. Marmisollé, Irene A. Keitelman, Analía S. Trevani, Sergio C. Oliveira, Omar Azzaroni, Paola L. Smaldini, Guillermo H. Docena

**Affiliations:** 1 Instituto de Estudios Inmunológicos y Fisiopatológicos (IIFP), Universidad Nacional de La Plata (UNLP), Consejo Nacional de Investigaciones Científicas y Técnicas (CONICET), Asociado a Comision de Investigaciones Cientificas de la Provicia de Buenos Aires (CIC PBA), Facultad de Ciencias Exactas, Departamento de Ciencias Biológicas, La Plata, Argentina; 2 Department of Biochemistry and Immunology, Institute of Biological Sciences, Federal University of Minas Gerais (ICB-UFMG), Minas Gerais, Brazil; 3 Instituto de Investigaciones Fisicoquímicas Teóricas y Aplicadas (INIFTA), (Universidad Nacional de La Plata (UNLP), Consejo Nacional de Investigaciones Científicas y Técnicas (CONICET)), La Plata, Buenos Aires, Argentina; 4 Instituto de Química de los Materiales, Ambiente y Energía (INQUIMAE), Universidad de Buenos Aires (UBA), Consejo Nacional de Investigaciones Científicas y Técnicas (CONICET), Facultad de Ciencias Exactas y Naturales, Departamento de Química Inorgánica Analítica y Química Física, Buenos Aires, Argentina; 5 Instituto para el Desarrollo Agroindustrial y de la Salud (IDAS), (Universidad Nacional de Rio Cuarto (UNRC), Consejo Nacional de Investigaciones Científicas y Técnicas (CONICET)), Río Cuarto, Argentina; 6 Laboratorio de Inmunidad Innata, Instituto de Medicina Experimental (IMEX), Consejo Nacional de Investigaciones Científicas y Técnicas (CONICET), Academia Nacional de Medicina, Buenos Aires, Argentina; 7 Department of Immunology, Institute of Biomedical Sciences, University of São Paulo (USP), São Paulo, Brazil

**Keywords:** adjuvant, coacervate, inflammasome, nanoparticle, NLRP3, systemic vaccines

## Abstract

Nanotechnology plays a crucial role in vaccine development, enabling the design of functional nanoparticles (NPs) that act as both antigen carriers and adjuvants to enhance immune responses. In this study, we evaluated complex coacervate-like NPs composed of poly(allylamine hydrochloride) (PAH) and tripolyphosphate (TPP) as a biocompatible and biosafe platform for systemic and mucosal subunit vaccines. We assessed NP-induced activation of antigen-presenting cells and their adjuvanticity in BALB/c and knockout mice immunized intraperitoneally and intranasally with NP-OVA. In vitro, NPs increased CD86 and MHC II expression and promoted interleukin-1β (IL-1β) and IL-18 secretion via NLRP3 inflammasome activation in macrophages and dendritic cells co-incubated with LPS. Cytokine release occurred through an unconventional autophagosome-dependent pathway, as inhibition of autophagy with 3-methyladenine reduced LPS/NP-induced IL-1β secretion. *In vivo*, NP-OVA administration induced robust OVA-specific IgG and IgG2a responses, increased IFN-γ secretion by splenocytes, and elevated frequencies of CD4⁺IFN-γ⁺ and CD8⁺IFN-γ⁺ T cells. Comparable responses were observed following intranasal immunization, highlighting the versatility of these NPs as vaccine platforms. Overall, PAH/TPP NPs activate the NLRP3 inflammasome in innate immune cells, promote antigen-presenting cell maturation, enhance cytokine secretion, and induce strong Th1-biased humoral and cellular immune responses. These findings support their potential as a safe dual-function nanoplatform for preventive and therapeutic vaccines against infectious and non-infectious diseases.

## 1 Introduction

Vaccination has prevented millions of deaths in recent decades, particularly in children, while reducing healthcare costs and enabling long-term coexistence with pathogens (1). Protein antigen-based vaccines have emerged as attractive vaccine platforms as they are safer than traditional vaccines (2). However, these formulations generally induce weak immune responses when administered alone (3). To overcome this limitation, adjuvants are incorporated to enhance protection by promoting robust and durable immunity (4). Adjuvants activate antigen-presenting cells (APCs), leading to the release of pro-inflammatory mediators and the clonal expansion of helper and cytotoxic T lymphocytes, which target and eliminate pathogen-infected cells and tumor cells (5, 6).

A further drawback of protein subunit vaccines is the susceptibility of protein- and peptide-based antigens to enzymatic degradation (7). Nanotechnology offers strategies for designing nanostructures that encapsulate, protect, and deliver antigens (3, 8, 9). For example, nanoparticle-based vaccines proved safe and effective during the COVID-19 pandemic (10–12). Additionally, some nanosystems function as self-adjuvants by enhancing immune responses (13–15). Several nanoparticles have been designed to stimulate innate immunity through the activation of the inflammasome in APCs (16–18). This internalization has been attributed to their size and morphology, which resemble those of pathogens (19–21).

Analogous to microbial- and damage-associated molecular patterns (MAMPs, PAMPs, and DAMPs), non-pathogenic nanoparticles can carry nanoparticle-associated molecular patterns (NAMPs) (19, 22). NAMPs engage pattern recognition receptors on the surface or inside cells, triggering inflammatory responses that activate immune cells (23, 24). Among these receptors, NLRP3, which is primarily expressed in myeloid cells like macrophages and dendritic cells, plays a key role in NAMP sensing. Nanoparticle-induced lysosomal disruption releases enzymes such as cathepsin B, which in turn activate the NLRP3 inflammasome (25). Upon activation of the inflammasome, the adaptor protein ASC assembles into large aggregates, which recruit pro-caspase and facilitates the conversion to its active form. This event drives the maturation of pro-inflammatory cytokines IL-1β and IL-18 and the pore-formin protein gasdermin-D. Gasdermin-D pores enable cytokine release but also damage the cell membrane, leading to pyroptosis, an inflammatory form of programmed cell death (26, 27). However, cytokines can be released through non-lytic mechanisms, including exocytosis or vesicle release, bypassing cell death (28, 29). For instance, multivesicular bodies, loaded with endosomes containing the cytokine, undergo lysis upon release into the extracellular environment, releasing soluble cytokines (30, 31). Additionally, alternative “non-canonical” inflammasome activation pathways involving caspase-11 have been described (32, 33).

A wide range of delivery systems has been explored as adjuvant/delivery platforms for subunit vaccines, including liposomes, PLGA nanoparticles, micelles, and lipid−polymer hybrid nanosystems (21, 34–38). Coacervation-like nanoplatforms based on ionic coacervation of polyamines (e.g., polyethyleneimine, chitosan, and polylysine) have also shown promise as adjuvants (39–41). These coacervate-based nanoformulations are typically engineered with a positive surface charge to facilitate uptake by APCs, which generally carry negatively charged membranes (7, 42). However, cationic systems often exhibit cytotoxicity due to disruptive interactions with cellular membranes (43, 44).

**Figure 1:**
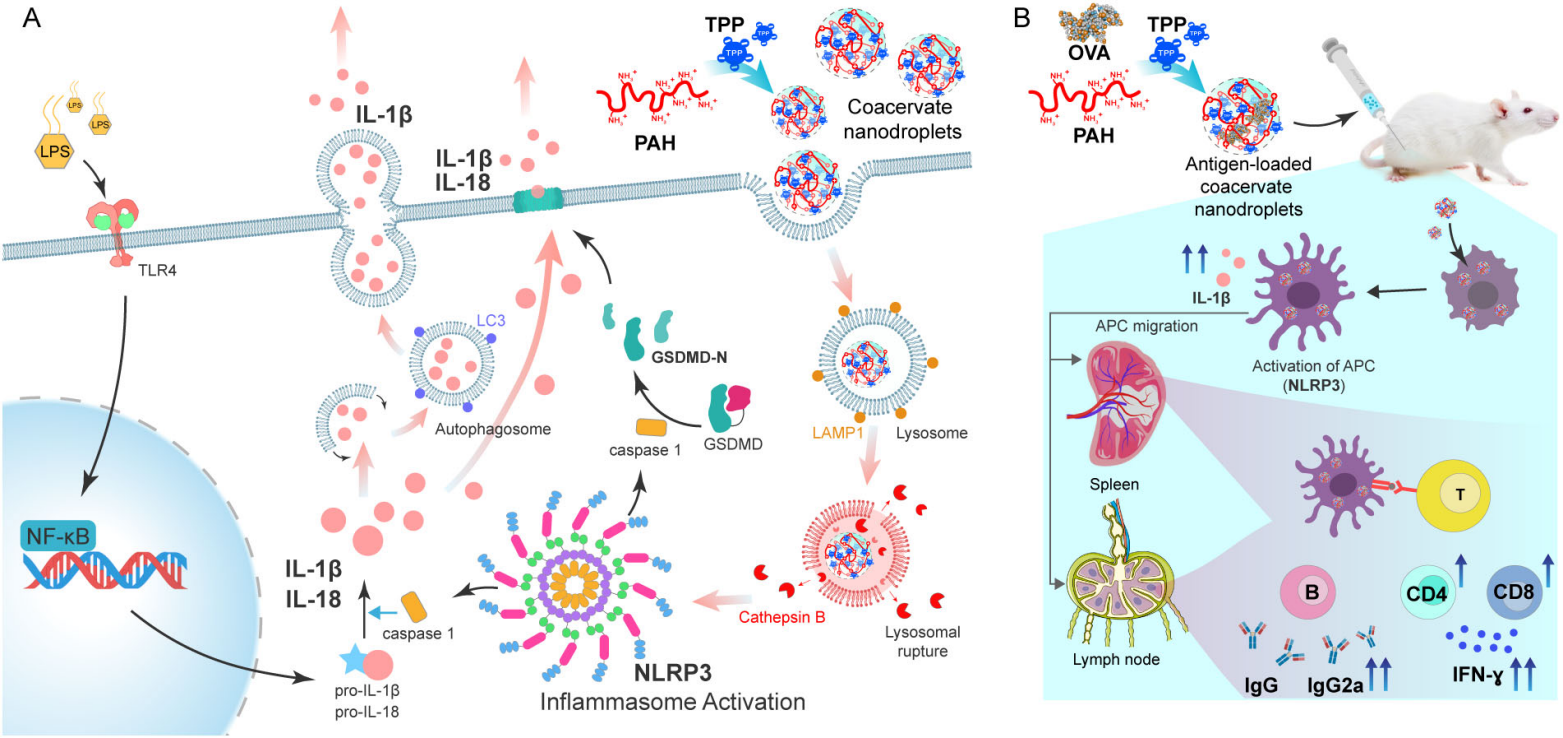
Mechanisms of NP-induced immune activation. **(A)** Schematic representation of NP uptake and NLRP3 inflammasome activation in phagocytic cells. Following NP internalization by phagocytosis, NPs traffic to lysosomes, where membrane destabilization leads to the release of cathepsin **(B)** Cathepsin B triggers the NLRP3 inflammasome pathway, resulting in caspase-1 activation, Gasdermin D cleavage, and maturation of pro-IL-1β and pro-IL-18 for secretion. Canonical inflammasome pathway activation requires a priming signal via Toll-like receptor to induce pro-cytokines expression. In the absence of pyroptosis, pro-inflammatory cytokines can alternatively be released through non-lytic mechanisms, including autophagy-associated pathways. **(B)** Systemic inoculation scheme with NP-OVA. Following uptake, phagocytic cells are activated and migrate to secondary lymphoid nodes, where they activate CD4+ and CD8+ T cells that produce IFN-γ. Activated CD4+ T cells provide help for B cell activation, leading to the generation of IgG and IgG2a antibodies.

We recently reported anionic coacervate nanoparticles (~200 nm in diameter and zeta potential ~ -30 mV) produced through aqueous assembly of polyallylamine (PAH) with the tripolyphosphate anion (TPP). These particles exhibited long-term stability, protein-encapsulation capacity, low cytotoxicity, and efficient uptake by macrophages and dendritic cells (45). Given the promising properties of PAH/TPP nanoparticles, here we explored their potential as adjuvants to enhance immune system activation. Encapsulation of an immunogen in this system may trigger innate immune cells and promote their migration to lymph nodes, where they can activate naïve B and T cells. This process is accompanied by cytokines and chemokines secretion and upregulation of key APC surface molecules, such as CD80, CD86, and MHC II, which potentiate T-cell activation (46–48). In this work, we demonstrate that dendritic cells and macrophages undergo activation upon nanoparticle uptake, driving Th1—type adaptive immunity. Specifically, NLRP3 inflammasome activation by PAH/TPP nanoparticles induced IL-1β and IL-18 release (**Figure 1A**), which in turn promoted the induction of both humoral and cellular immune responses, through the induction of CD4^+^- and CD8^+^-IFN-γ-producing cells, IFN-γ secretion, and antigen-specific IgG production (**Figure 1B**). Collectively, our findings indicate that the PAH/TPP coacervate nanoformulation functions as both safe antigen carriers and nano-adjuvants, offering a promising platform for next-generation vaccine development.

## 2 Materials and methods

### 2.1 Preparation and characterization of PAH/TPP nanoparticles

PAH/TPP nanoparticles were prepared as described by Apuzzo et al. (49). Briefly, $0.125\;V_{f}$ ml of PAH (17.5 kDa, Sigma-Aldrich, St. Louis, MO, USA) at 40 mM (monomer concentration, pH 8.5), $0.75\;V_{f}$ ml of deionized water, and $0.125\;V_{f}$ ml of TPP (Sigma-Aldrich, St. Louis, MO, USA) at 24 mM (pH 8.5) were mixed under constant stirring at room temperature (RT). TPP was added rapidly to prevent the system from collapsing into a macroscopic phase. This procedure yielded a final dispersion of PAH/TPP nanoparticles with 5 mM PAH and 3 mM TPP. In this work, NP concentration was expressed in terms of PAH monomer concentration (i.e., 0.5 mM NP corresponds to 1:10 dilution of the stock dispersion). For protein-loaded nanoparticles, the same protocol was followed, except that an aqueous solution of chicken egg albumin or ovalbumin (OVA) (EndoFit^(TM)^ InvivoGen, San Diego, CA, USA) was added during the water addition step. The volume of deionized water was adjusted as $V_{water}=0.75\;V_{f}-V_{OVA}$ and the OVA volume was fixed to obtain the desired final OVA concentration. After synthesis, NP-OVA was incubated at room temperature for 1 h and then centrifuged at 14,000 × g for 20 min. Under these conditions, PAH/TPP NPs are expected to efficiently sediment, forming a pellet, while non-encapsulated protein remains in the supernatant. This assumption was supported by DLS measurements, which showed negligible nanoparticle signal in the supernatant after centrifugation. Therefore, encapsulation efficiency was determined by quantifying protein in the supernatant using a bicinchoninic acid (BCA) assay kit (Thermo Fisher Scientific, Waltham, MA, USA).

For nanoparticle imaging, atomic force microscopy (AFM) was performed. Samples were imaged with an Agilent 5500 SPM in tapping mode at room temperature using PPP-NCL (NANOSENSORS™) silicon tips. A droplet of PAH/TPP NP suspension was placed on top of a freshly cleaved highly oriented pyrolytic graphite (HOPG) substrate, incubated for 2 min, rinsed with Milli-Q water, and dried with N_2_. For transmission electron microscopy (TEM) images were acquired with a JEOL microscope (120 kV) equipped with a Gatan US1000 CCD camera. For sample preparation, 5 µl of a PAH/TPP NP dispersion was placed on carbon-coated copper grid and stained with phosphotungstic acid to create image contrast. Dynamic light scattering (DLS) and ζ-potential measurements were performed using a ZetaSizer Nano (ZEN3600) at 20 °C with disposable cuvettes. Hydrodynamic diameter was measured using a 173° backscatter angle with 20 runs per sample, averaging 5 measurements. ζ-potential was calculated from electrophoretic mobility with 100 runs per sample. Confocal (epifluorescence) microscopy of fluorescent PAH/TPP NPs was carried out using a Carl Zeiss Axio Observer 7 with a Plan-Apochromat 63x/1.40 Oil DIC M27 objective.

### 2.2 Cell lines and culture conditions

HEK-hTLR4-reporter (human embryonic kidney cells stably transfected with human TLR4 and an NF-κB-inducible reporter gene; InvivoGen^®^, San Diego, CA, USA), HT-29 (human colorectal adenocarcinoma epithelial cell line) and J774 (mouse macrophage cell line derived from BALB/c reticulum sarcoma) cell lines were cultured in DMEM medium (Gibco^®^ Thermo Fisher Scientific, Waltham, MA, USA) supplemented with 10% FBS (Internegocios, Mercedes, Bs As, Argentina), 100 U/mL penicillin and 100 µg/mL streptomycin (Gibco^®^ Thermo Fisher Scientific, Waltham, MA, USA). The THP1-ASC-GFP cells (human monocytic leukemia cell line THP-1 stably transfected with ASC-GFP reporter; InvivoGen^®^) were maintained in RPMI 1640 medium (Gibco^®^ Thermo Fisher Scientific, Waltham, MA, USA) containing 10% FBS, 100 U/mL penicillin and 100 µg/mL streptomycin.

Bone marrow-derived macrophages (BMDMs) and bone marrow-derived dendritic cells (BMDCs) were obtained from wild-type (WT) and knockout (KO) mice (see below for specific genotypes). BMDMs were cultured in DMEM containing 20% L929-conditioned medium (LCCM), while BMDCs were cultured in RPMI medium supplemented with 20 ng/mL recombinant granulocyte-macrophage colony-stimulating factor (GM-CSF) (R&D Systems, Minneapolis, MN, USA). Briefly, bone marrow cells were isolated from femurs and tibias of WT or KO mice, seeded at 5x10^5^ cells/mL (day 0) and cultured in a conditioned medium and GM-CSF containing 10% FBS, 100 U/mL penicillin, and 100 µg/mL streptomycin at 37 °C with 5% CO_2_ atmosphere for 7 days. Medium was replaced on day 4, and cells were stimulated at day 7 with 0.5 mM PAH/TPP NPs. For positive control for inflammasome activation, cells were primed with 1 μg/mL LPS (*E. coli*, Sigma-Aldrich, St. Louis, MO, USA) for 3 h at 37 °C, followed by 5 mM Nigericin for 50 min. Supernatants and cell lysates, prepared with 25 µL/well M-PERR (Thermo Fisher Scientific, Waltham, MA, USA) and supplemented with 1:100 protease inhibitor cocktail (Sigma-Aldrich, St. Louis, MO, USA) were stored at −80 °C.

### 2.3 Cell stimulation with nanoparticles and functional assays

#### 2.3.1 Flow cytometry for phenotype and cell activation characterization

BMDCs were seeded in 24-well microplates (1x10^6^ cells/well), primed with ultrapure LPS from *E. coli* (1 µg/mL, 3 h, 37 °C), and then stimulated overnight (ON) with 0.5 mM PAH/TPP NPs. Cells were washed with PBS and stained for 1 h at 4 °C with anti-CD11c (PE), anti-CD11b (PerCP-Cy5.5), anti-MHCII (APC), and anti-CD86 (FITC) (Thermo Fisher Scientific, Waltham, MA, USA). As positive control, cells were primed with ultrapure LPS (1 µg/mL, 3 h) and stimulated with 5 mM ATP (Sigma-Aldrich, St. Louis, MO, USA) for 3 h at 37 °C. Fluorescence was acquired on a FACS Aria Fusion cytometer and analyzed with FlowJo.

#### 2.3.2 Reagents for cell activation suppression

For inhibition assays, cells were pre-treated for 30 min with 100 µg/mL Z-VAD-FMK, 1 µg/mL cytochalasin D or 50 µM CA-74ME, 5 mM 3-methyl adenine (Cayman, Ann Arbor, MI, USA) and 1 µM VPS34-IN1 (Cayman, Ann Arbor, MI, USA) or 100 nM Bafilomycin A1 (Cayman, Ann Arbor, MI, USA), and maintained in culture throughout the experiment. When indicated, at 4 h post-NP stimulation, supernatants were replaced with either complete medium or Earle’s Balanced Salt Solution (EBSS) for nutrient starvation.

#### 2.3.3 Nanoparticle internalization analysis

BMDCs and BMDMs were cultured on glass coverslips and stimulated ON with 0.5 mM FITC-PAH/TPP NP. Poly(fluorescein isothiocyanate allylamine hydrochloride), Mw~56000 (Sigma-Aldrich, St. Louis, MO, USA) was used instead of PAH in the PAH/TPP formulation. Cells were fixed in 4% paraformaldehyde (PFA) for 30 min at RT, and nuclei were stained with 1 µg/mL propidium iodide (PI) (Sigma-Aldrich, St. Louis, MO, USA). Cells were washed with PBS, blocked with 2% BSA in phosphate buffer (pH=7.4) for 30 min at RT, and incubated with mouse anti-human LAMP1 (BD Biosciences, Franklin Lakes, NJ, USA) for 1 h at RT, followed by Alexa Fluor 647-conjugated anti-mouse IgG (Abcam, Cambridge, UK) for 1 h at RT. Cells were washed, coverslips were mounted onto a slide containing 10 µL of fluorescence mounting media (Dako, Abcam, Cambridge, UK) and dried ON at 4 °C. Images were analyzed using a Leica SP5 confocal microscope.

#### 2.3.4 Internalization inhibition assay

BMDCs and J774 macrophages were stimulated with 0.5 mM FITC-PAH/TPP NPs, and phagocytosis was inhibited with 1 µg/mL cytochalasin D. Cells were characterized by flow cytometry using anti-CD11b (Percp-Cy5.5) and anti-F4/80 (APC) (Thermo Fisher Scientific, Waltham, MA, USA), and by confocal microscopy to detect intracellular fluorescence. Nuclei were stained with 1 µg/mL PI.

#### 2.3.5 Intracellular immunostainings for the localization of IL-1β in autophagosomes

J774 cells were cultured on glass coverslips and primed for 3 h at 37 °C with 1 µg/mL LPS from E. coli, followed by stimulation with 0.5 mM PAH/TPP NP for 6 h. Cells were fixed with 4% PFA for 30 min, blocked with PBS-glycine (0.1 M) for 15 min, permeabilized with acetone (−20 °C, 7 min), and blocked with 5% PBS-BSA for 1 h at RT. Macrophages were then incubated ON at 4 °C with the rabbit polyclonal anti-LC3B (Proteintech, Rosemont, IL, USA) and mouse polyclonal anti-IL-1β (Cell Signaling Technology, Danvers, MA, USA) in blocking buffer, followed by Alexa Fluor 594-conjugated anti-rabbit IgG (Abcam, Cambridge, UK) and Alexa Fluor 488-conjugated anti-mouse IgG (Invitrogen, Thermo Fisher Scientific, Waltham, MA, USA) for 1 h at RT. Nuclei were stained with methyl green (Sigma-Aldrich, St. Louis, MO, USA) and mounted onto a slide containing fluorescence mounting medium (Dako, Abcam, cambrige, UK). Images were acquired using a SP5 Leica confocal microscope.

#### 2.3.6 Epithelial cell stimulation

HT-29 cells were maintained in DMEM supplemented with 10% FBS, 100 U/mL penicillin, and 100 µg/mL streptomycin at 37 °C with 5% CO_2_. Cells (3x10^4^/well) were seeded in 96-well, incubated ON to reach 70% confluence, after which the medium was replaced. Cells were then stimulated ON with 0.5 mM FITC-PAH/TPP NP, or pre-treated with 50 ng/mL TNF-α (R&D Systems, Minneapolis, USA) for 30 min at 37 °C before being stimulated with FITC-PAH/TPP NP. For epithelial cell detection, a mouse monoclonal anti-human EPCAM antibody (Santa Cruz Biotechnology, Santa Cruz, CA, USA), followed by an Alexa Fluor 647-conjugated anti-mouse antibody IgG (Abcam, Cambridge, UK), was used. Fluorescence was analyzed with a Leica SP5 confocal microscope.

#### 2.3.7 Inflammasome activation assay

THP1-ASC-GFP cells were maintained in RPMI medium supplemented with 10% FBS, 100 U/mL penicillin, and 100 µg/mL streptomycin (37 °C, 5% CO_2_), and plated at 3.6×10^5^ cells/well in 96-well plates. Cells were stimulated with 0.5 mM PAH/TPP NP or primed with 1 µg/mL ultrapure LPS from *E. coli* followed by 0.5 mM PAH/TPP NP ON. As a positive control, cells were primed with 1 µg/mL LPS from *E. coli* for 3 h and transfected with 500 ng/mL bacterial plasmid cDNA. pcDNA was generated by cloning the PCR-generated full-length cDNA from a random non-related gene. Transfection was carried out with Lipofectamine™ LTX Reagent and PLUS TM Reagent (Invitrogen, Thermo Fisher Scientific, Waltham, MA, USA), following the manufacturer´s instructions. Supernatants were harvested and stored at −80 °C. Cells were stained with DAPI (Invitrogen, Thermo Fisher Scientific, Waltham, MA, USA) for nuclear visualization and fixed with 2% PFA for microscopy.

The frequency of ASC-GFP^+^ cells and the localization of fluorescent ASC specks were assessed using a Nikon Eclipse Ti fluorescence microscope and analyzed with ImageJ software. The total cells: ASC-GFP^+^ cells ratio was calculated.

#### 2.3.8 Quantification of secreted cytokines

Supernatants of stimulated BMDMs, J774, BMDCs, THP1, THP1-ASC-GFP and HT-29 cells were assessed for mouse (m) IL-1β, mIL-18, mIL-6, mIFN-γ and mTNF-α using Mouse DuoSet R&D Systems ELISA kit. Levels of human (h) IL-1β and hIL-8 were measured with human ELISA Kits (Invitrogen, Thermo Fisher Scientific, Waltham, MA, USA). LDH release was quantified using an enzymatic assay (LDH-L Weiner Lab, Rosario, Santa Fe, Argentina). All protocols were used according to the manufacturer’s instructions.

#### 2.3.9 Immunoblotting

Cell lysates and supernatants were analyzed by SDS-PAGE and immunoblotting. Proteins loading buffer (SDS+β-mercaptoethanol) were separated on 15% SDS-PAGE gels and transferred onto nitrocellulose membranes (Amersham Biosciences, Uppsala, Sweden) using transfer buffer (50 mM Tris, 40 mM glycine, 10% methanol). Membranes were blocked for 1 h in TBS with 0.1% Tween-20 and 5% non-fat dry milk, then incubated ON at 4 °C with mouse monoclonal anti-caspase-1 p20 (Adipogen, San Diego, CA, USA) (1:1000). A loading control was performed with an anti–β-actin monoclonal antibody (Cell Signaling Technology, Danvers, MA, USA) (1:1000). After washing, membranes were incubated for 1 h at RT with HRP-conjugated secondary antibody (Cell Signaling Technology, Danvers, MA, USA) (1:1000). Immunoreactive bands were visualized using Luminol chemiluminescent HRP substrate (Millipore, Burlington, MA, USA) and analyzed with ImageQuant TL Software (GE Healthcare, Buckinghamshire, UK).

### 2.4 LPS content of PAH/TPP nanoparticles

The HEK-hTLR4 reporter cells were maintained in DMEM supplemented with 10% FBS, 100 U/mL penicillin, and 100 µg/mL streptomycin (37 °C, 5% CO_2_). A total of 3 x10^4^ cells/well were seeded in 96-well plates and incubated to 70% confluence. The medium was replaced, and cells were stimulated ON with different concentrations of LPS, 0.5 mM PAH, 0.3 mM TPP, or 0.5 mM PAH/TPP NP. Supernatants were collected, and secreted embryonic alkaline phosphatase (SEAP) activity was measured.

### 2.5 *In vivo* assessment of adjuvant properties

#### 2.5.1 Inflammasome characterization

Male 6-9-week-old wild-type and knockout (KO) C57BL/6 mice (Nlrp3^-/-^, Casp1/11^-/-^, Casp11^-/-^ and Gsdmd^-/-^) were purchased from the Federal University of Minas Gerais and maintained under 12-h light/dark cycles. Femurs and tibiae were collected for BMDM preparation. In addition, male wild-type BALB/c mice (5–7 weeks old) were purchased from the Laboratory of Experimental Animals, School of Veterinary Sciences of the University of La Plata (UNLP), Argentina.

Wild-type C57BL/6 and BALB/c mice, as well as KO C57BL/6 mice, were immunized once per week for three consecutive weeks. For intraperitoneal (IP) administration mice received 100 µg OVA-conjugated PAH/TPP nanoparticles (NP-OVA; 2.5 mM in 500 µL) or 100 µg OVA adjuvanted with 500 µg of aluminum hydroxide (1 mg/mL in 500 µL PBS) (OVA+Alum). Control animals received 100 µg OVA EndoFit^(TM)^ alone in 500 µL. For intramuscular (IM) administration 100 µg OVA or NP-OVA was injected in 50 µL into the quadriceps muscle of the hind leg. For intranasal (IN) administration 100 µg NP-OVA and OVA control was delivered in 20 µL (10 µL per nostril) to mice anesthetized with isoflurane. One week after the final immunization, mice were bled and euthanized for immune response analysis.

#### 2.5.2 Humoral response assessment

OVA-specific antibodies were quantified by indirect ELISA in different biological samples. Briefly, MaxiSorp plates (NUNC, Roskilde, Denmark) were coated with 1 µg/mL OVA, 5 mM PAH or 5 mM NP in carbonate-bicarbonate buffer (pH=9.6). Plates were blocked with 5% equine serum in saline for 1 h at 37 °C, followed by incubation with sera (1:100) for 1 h at 37 °C. Bound immunoglobulins were detected using anti-mouse IgG-HRP (Biorad, Hercules, CA, USA), anti- mouse IgG1-HRP or anti-mouse IgG2a-HRP (BD Biosciences, Franklin Lakes, NJ, USA). The reaction was developed with TMB (Sigma-Aldrich, St. Louis, MO, USA) and stopped with 2 M H_2_SO_4_. OD was measured at 450 nm with Varioskan Lux plate reader (Thermo Fisher Scientific).

#### 2.5.3 Cytokine production in stimulated splenocytes

Splenocyte suspensions were prepared in RPMI-1640 supplemented with 10% FBS, 100 U/mL penicillin, and 100 µg/mL streptomycin, seeded (4x10^6^ cells/mL) in culture plates, and cultured for 72 h at 37 °C under controlled conditions. Cells were restimulated with 10 μg/mL OVA, and supernatants were collected for quantification of mIFN-γ, mIL-5, and mIL-13 by ELISA using commercial kits (R&D Systems, Minneapolis, MN, USA), according to the manufacturer's instructions.

Intracellular cytokines were analyzed by flow cytometry. Splenocytes were incubated with brefeldin A (BD Biosciences, Franklin Lakes, NJ, USA) for the final 4 h of culture, and stained with anti-CD4 (PerCP-Cy5.5) and anti-CD8 (APC) monoclonal antibodies (Thermo Fisher Scientific, Waltham, MA, USA). After fixation and permeabilization, cells were stained with a FITC-conjugated anti-mouse IFN-γ antibody (Thermo Fisher Scientific, Waltham, MA, USA). Samples were acquired on a FACS Aria Fusion cytometer, and data were analyzed using FlowJo software.

#### 2.5.4 Ethics

All experimental procedures were conducted in compliance with international standards for animal experimentation (Helsinki Declaration and its amendments, Amsterdam Protocol of Welfare and Animal Protection and National Institutes of Health, USA NIH, guidelines: Guide for the Care and Use of Laboratory Animals). Anesthetized mice (isoflurane 5%) were euthanized by cervical dislocation performed by trained personnel. Every effort was made to alleviate animal suffering. All protocols were approved by the Institutional Committee for the Care and Use of Laboratory Animals of the School of Sciences (University of La Plata) (Protocol Number: 006-37-21).

### 2.6 Statistical analysis

All experiments were repeated in duplicate with consistent results. Flow cytometry data were analyzed using FlowJo X, and microscopy images were analyzed with ImageJ. Graphs and statistical analyses were performed with GraphPad Prism8. Statistical comparisons were conducted using one- or two-way ANOVA followed by Tukey´s test or Student´s-test when applicable. Quantitative results are expressed as mean ± SEM. Statistical significance was defined as p<0.05.

## 3 Results

### 3.1 Nanoparticle preparation and characterization

**Figure 2:**
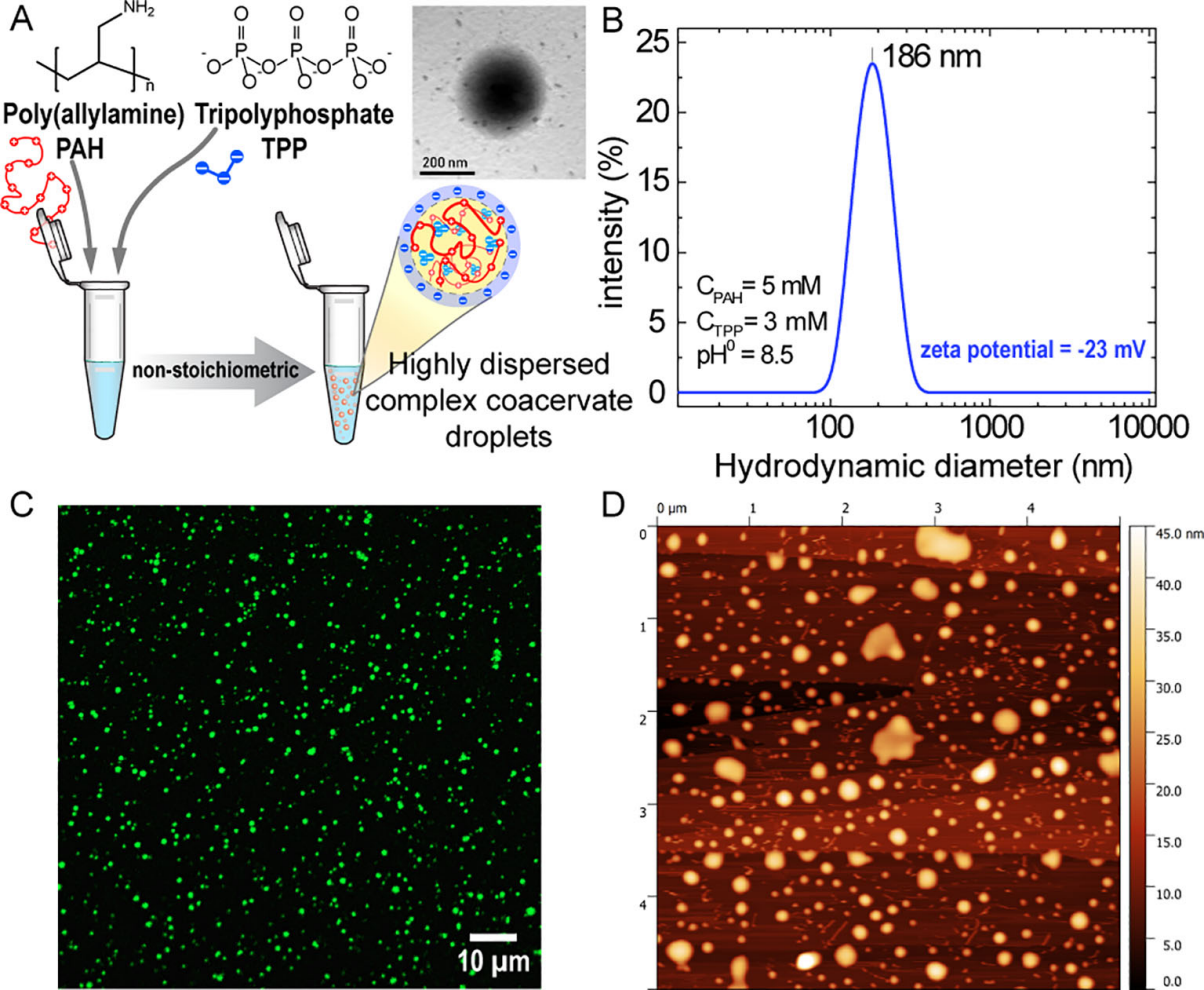
Characterization of PAH/TPP nanoparticles (NPs). **(A)** Schematic of PAH/TPP NP synthesis. Mixing 5 mM PAH (monomer concentration) with 3 mM TPP in aqueous solution yields a stable colloidal dispersion of asymmetric coacervate droplets stabilized by electrostatic repulsions. The inset shows a TEM image of a single PAH/TPP NP. **(B)** Dynamic light scattering (DLS) analysis showing a monodisperse particle population with a mean hydrodynamic diameter of 186 nm. **(C)** Confocal microscopy image of a coverslip exposed for 2 min to a PAH*/TPP NP dispersion. **(D)** Atomic force microscopy (AFM, tapping mode) topography image of a highly oriented pyrolytic graphite (HOPG) substrate after 2 min exposure to a PAH/TPP NP dispersion.

The interaction of polyamines with negatively charged multivalent small molecules has been thoroughly studied over the past years. Upon mixing these components in aqueous solution, phase condensation occurs, leading to the formation of asymmetric polyelectrolyte complexes that contain both intrinsic and extrinsic ion pairs between the long-chain polycation and short-chain polyanions (50, 51). Under charge-matching conditions (i.e., when the total amount of negative charges equals the total amount of positive charges), mixing polyamines with polyphosphates typically yields macroscopic phases, ranging from insoluble solids (highly crosslinked polyelectrolyte complexes) to polyelectrolyte-rich immiscible liquids (complex coacervates), depending on pH and monovalent salt concentration. We recently reported the formation of an asymmetric coacervate composed of PAH (long-chain polyelectrolyte block) and TPP (small multivalent molecule) (45). When the mixture is prepared under highly non-stoichiometric conditions and in the absence of added monovalent salts, the colloidal dispersion of coacervate droplets remains stable and does not collapse into a macroscopic liquid phase, as usually observed for symmetric coacervates prepared under stoichiometric conditions. **Figure 2A** shows a simplified scheme of the preparation process, in which PAH chains are ionically crosslinked by TPP anions, giving rise to nanometric-sized coacervate droplets (nanocomplexes). For clarity, these nanocomplexes will hereafter be referred to as nanoparticles (NPs).

Through dynamic light scattering (DLS) analysis, we detected a single particle size distribution with a mean hydrodynamic diameter near 200 nm (**Figure 2B** and TEM) and a ζ-potential of -23 mV. These NPs remained stable in colloidal dispersion for more than 9 months when stored in a closed vessel (45), a stability attributed to their negative surface charge of NPs resulting from the excess of TPP anions. Also, we demonstrated that using a ratio of concentrations C_TPP_/C_PAH_=0.6, the excess of negative charges forced approximately 95% of PAH chains (~95%) to be ionically crosslinked, leaving no detectable free PAH in solution (45). Using FITC-labeled PAH (PAH*), confocal microscopy confirmed that PAH*/TPP NPs displayed a homogeneous size distribution (**Figure 2C**). Finally, atomic force microscopy (AFM) analysis further revealed that upon contact with a flat surface such as highly oriented pyrolytic graphite (HOPG), NPs deformed by expanding in diameter and compressing in height, demonstrating their viscous nature (**Figure 2D**).

### 3.2 Nanoparticle internalization triggers activation in phagocytic cells

**Figure 3:**
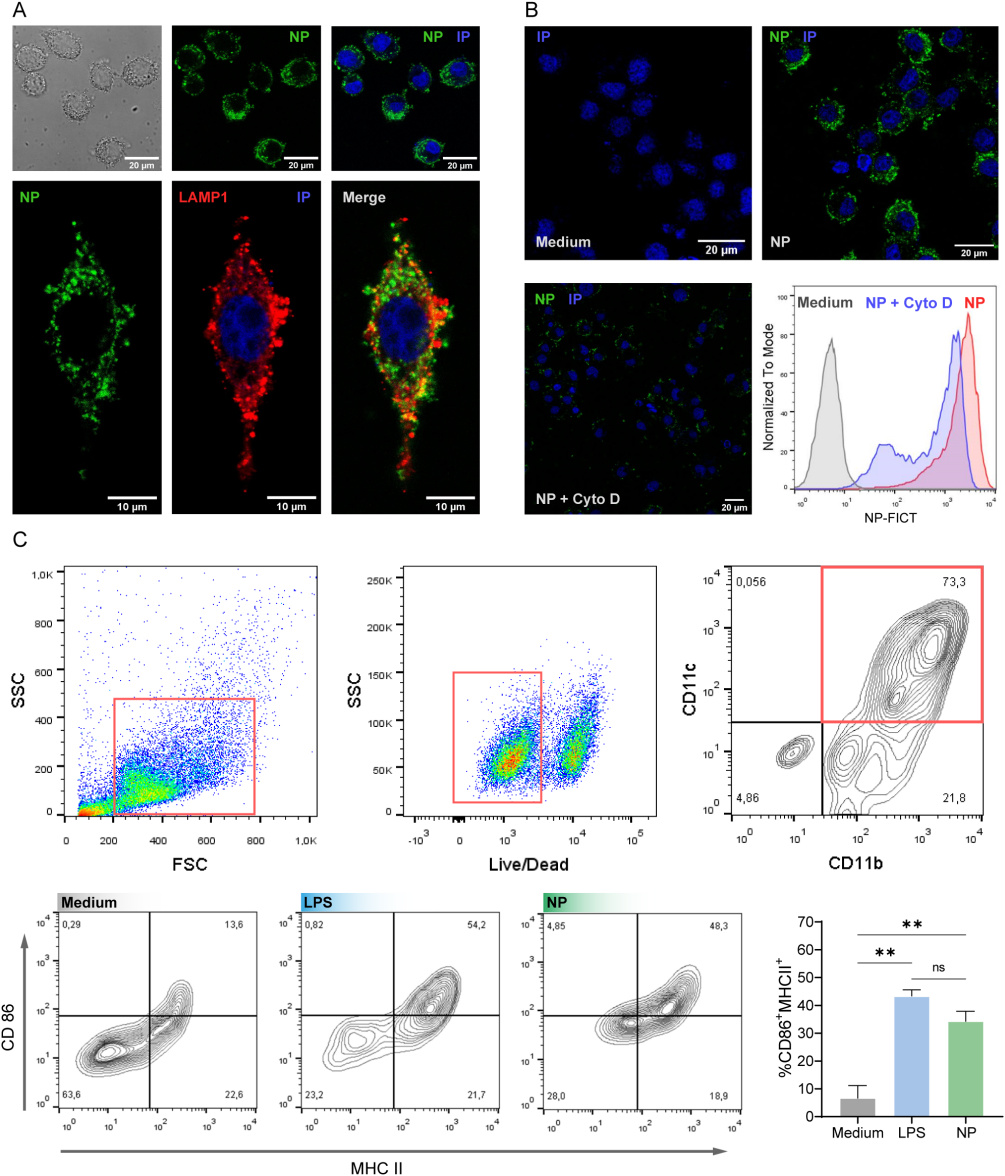
Cellular uptake and activation induced by PAH/TPP nanoparticles (NPs). **(A)** Confocal microscopy showing internalization of FITC-labeled NPs (NP-FITC) by J774 and colocalization with the late endosomal marker LAMP-1 after 4 h of stimulation. **(B)** Inhibition of NP-FITC phagocytosis in bone-marrow-derived macrophages (BMDM) by cytochalasin D, assessed by confocal microscopy and flow cytometry. **(C)** Flow cytometry analysis of surface expression of CD86 and MHC II on BMDC (CD11b^+^CD11c^+^). All experiments were performed in triplicate. Data are presented as mean ± SEM. Statistical significance was determined by One-way ANOVA (**P<0,01). ns, not significant.

To study the interaction of PAH/TPP NPs with cells, we exposed the murine J774 macrophages to NP-FITC. Confocal microscopy after 4 h of exposure revealed efficient internalization and cellular distribution of fluorescent NPs, whereas free PAH-FITC (without TPP) was not internalized (**Supplementary Figure S1**). Internalized NPs appeared both as fluorescent granules within cytoplasmic vesicles and as diffuse fluorescence in the cytosol (**Figure 3A**). Co-localization analysis with the late endosomal marker LAMP1 showed that NPs were distributed between LAMP1^+^- and LAMP1^--^vesicles, suggesting localization in both late and early endosomes (yellow and green vesicles, respectively). Treatment with the phagocytosis inhibitor cytochalasin D (Cyto D) confirmed that NP uptake was phagocytosis-dependent. Macrophages incubated with Cyto D exhibited reduced fluorescence intensity, as determined by flow cytometry in F4/80^+^ CD11b^+^ cells (**Figure 3B**; **Supplementary Figure S1C**). Next, bone marrow-derived dendritic cells (BMDC) were exposed to NP or LPS (positive control). Flow cytometry analysis revealed significantly increased expression of Class II MHC and CD86 in NP- or LPS-treated BMDC (**Figure 3C**). The magnitude of Class II MHC and CD86 upregulation was comparable between NP- and LPS-stimulated BMDCs, indicating that PAH/TPP NPs efficiently activated dendritic cells.

To test whether intestinal epithelial cells could also internalize NPs, we incubated the human intestinal epithelial cell line HT-29 with NP-FITC. Confocal microscopy using EPCAM as an epithelial marker, together with z-series imaging, showed no evidence of NP internalization. Moreover, neither basal nor TNF-α-pre-treated HT-29 cells (20 ng/mL) produced detectable hIL-8 or showed intracellular fluorescence upon NP exposure *in vitro* (**Supplementary Figures S2A, B**). We then investigated the NP-induced secretion of mIL-1β in a phagocytic cell line. We exposed cells to LPS (1 μg/mL) as a prime stimulus, followed by a dose-response incubation with NPs. The highest secretion of mIL-1β was observed at 0.5 mM NP (expressed as PAH monomer concentration), indicating that PAH/TPP NPs can trigger mIL-1β release in phagocytic cells (**Supplementary Figure S2C**).

**Figure 4:**
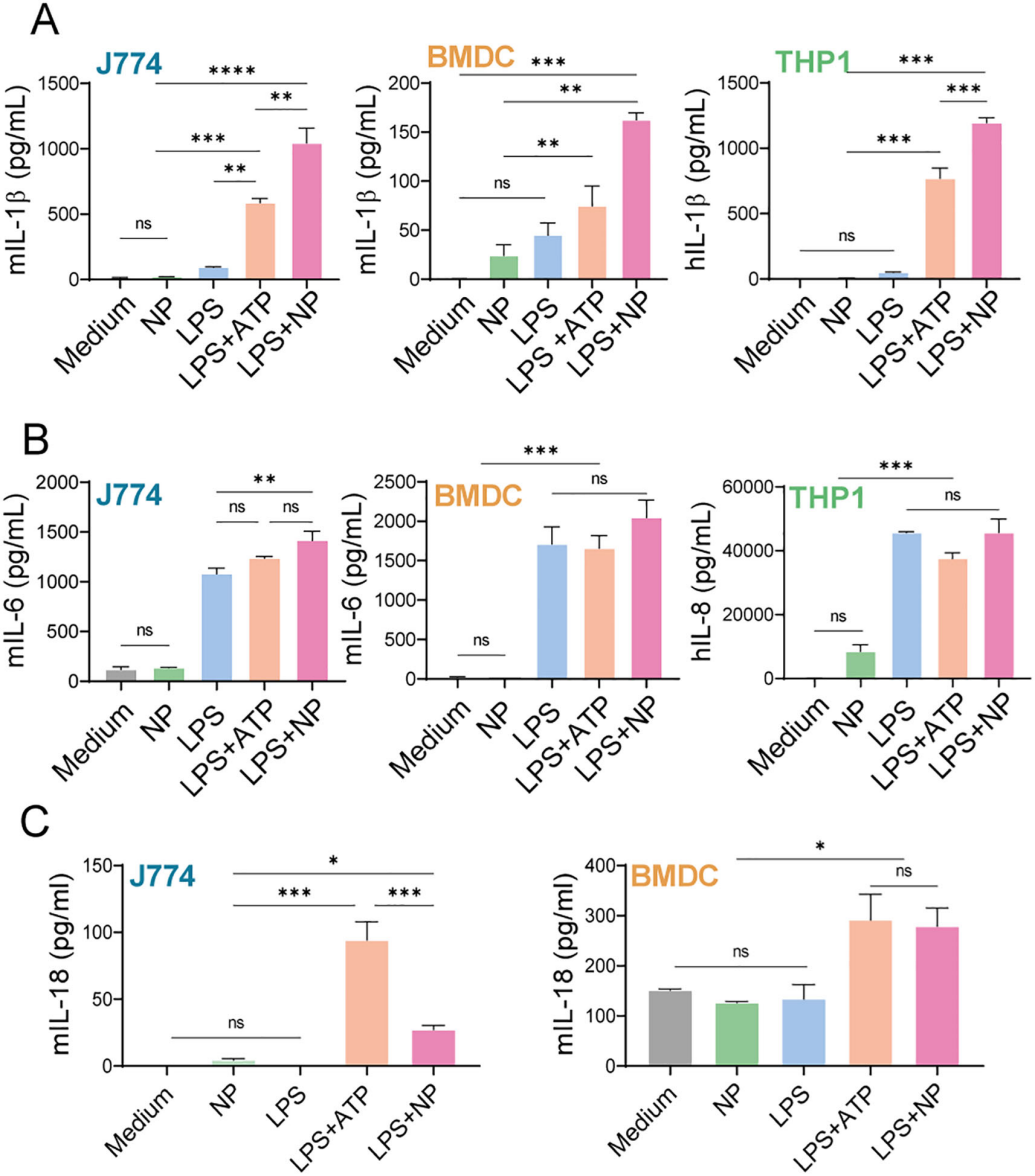
Secretion of pro-inflammatory cytokine by macrophages, dendritic cells and monocytes. J774, THP-1, and BMDCs were primed with LPS (1 μg/mL, 3 h), after which the medium was replaced and cells were stimulated overnight with PAH/TPP NPs (0.5 mM) or for 3 h with ATP (50 nM, positive control). Additional controls included cells incubated with LPS or NP alone. Cytokine secretion was quantified in culture supernatants by ELISA **(A)** IL-1β and **(B)** IL-6 and IL-8 and **(C)** IL-18. All experiments were carried out in triplicate. Data are expressed as mean ± SEM. Statistical significance was determined by One-way ANOVA (*P<0,05; **P<0,01; ***P<0,001; ****p<0,0001; ns, not significant).

We next investigated the production of pro-inflammatory cytokines by phagocytic cells exposed to 0.5 mM NP or controls (**Figure 4**). Murine J774 macrophages, BMDCs, and human THP-1 monocytes secreted significantly higher levels of IL-1β when stimulated with LPS+NP compared with LPS or NP alone (**Figure 4A**). NP+LPS rendered increased levels of IL-1β compared with LPS+ATP, a strong NLRP3 activator as a positive control (52). These findings collectively suggest that NP-induced IL-1β secretion required LPS priming. Analysis of mIL-6 and hIL-8 revealed no significant changes in cytokine levels under LPS or LPS+NP conditions, and NP treatment alone resulted in basal secretion (**Figure 4B**). By contrast, mIL-18 secretion was selectively enhanced in J774 macrophages and BMDCs incubated with LPS+NP. Cells exposed to medium, LPS or NPs alone remained at baseline. Both LPS+ATP and LPS+NP induced the highest mIL-18 levels in BMDCs (**Figure 4C**). Together, these findings demonstrate that PAH/TPP NPs cooperate with LPS priming to induce IL-1β and IL-18 secretion in APCs, while not affecting IL-6 or IL-8 production.

### 3.3 Nanoparticles activate the intracellular inflammasome pathway

**Figure 5:**
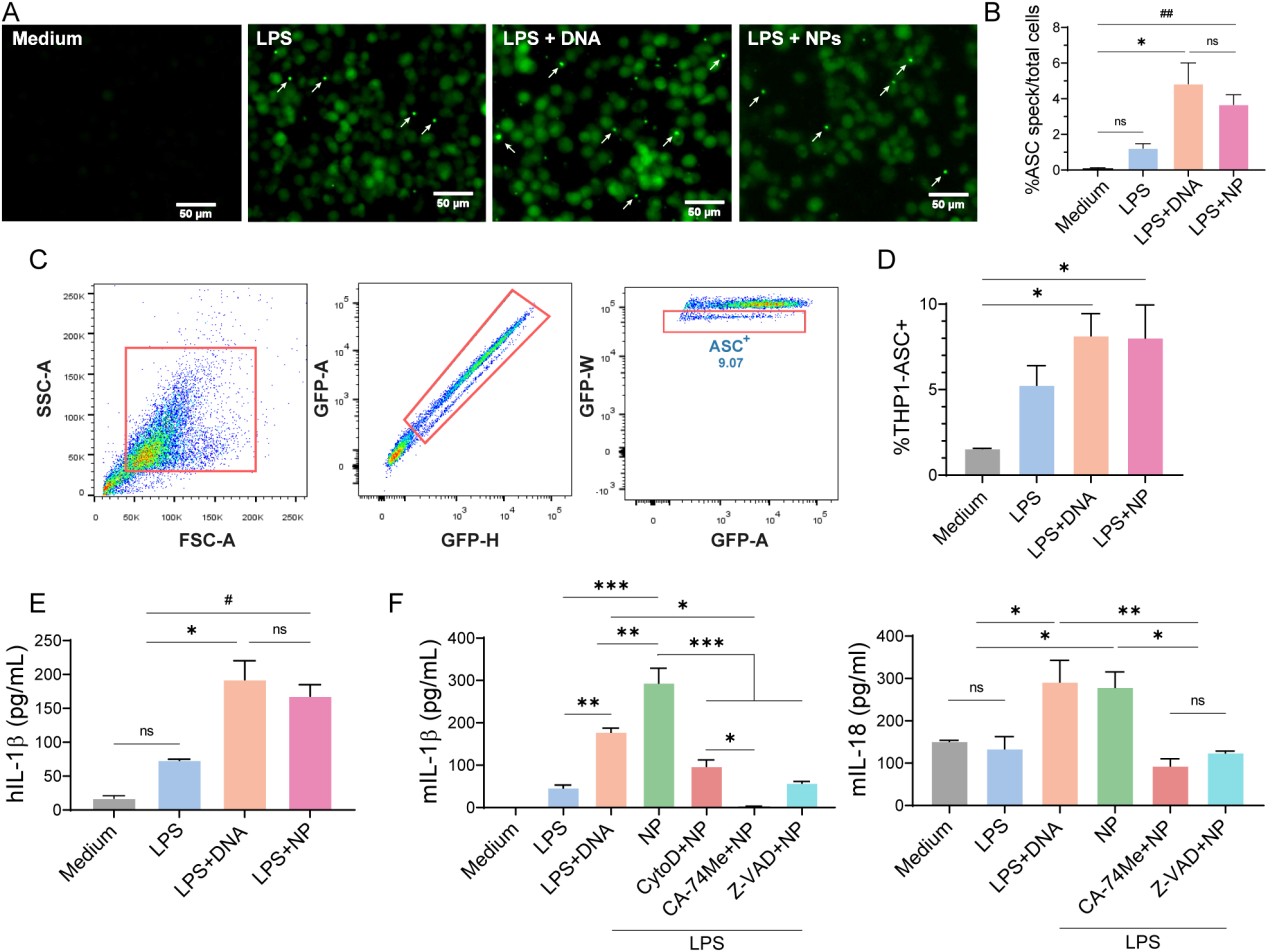
Inflammasome activation and cytokine secretion in THP1 ACS-GFP cells and BMDCs. THP1 ACS-GFP cells and BMDCs were primed with LPS for 3 h, after which the medium was replaced. Cells were then stimulated overnight with PAH/TPP NPs or transfected with pcDNA (positive control). **(A)** Epifluorescence microscopy showing ASC**-**GFP speck formation (white arrow); **(B)** Quantification of speck- ASC-GFP^+^cells. Data represent the mean number of speck^+^ cells per total cells per field from three independent experiments, three fields per treatment were counted (two replicates per treatment); **(C)** Flow cytometry gating for ASC-GFP^+^cells; **(D)** Frequency of speck ASC-GFP^+^cells by flow cytometry; **(E)** Quantification of secreted hIL-1β by ELISA. **(F)** BMDCs were pre-incubated for 30 min with cytochalasin D (phagocytosis inhibitor), CA-74Me (cathepsin B inhibitor), and Z-VAD-FMK (pan-caspase inhibitor) prior to overnight stimulation with LPS+NP. The levels of secreted mIL-1β and mIL-18 were quantified by ELISA. All experiments were carried out in triplicate. All experiments were performed in triplicate. Data are expressed as mean ± SEM. Statistical significance was determined by One-way ANOVA (*p<0,05; **p<0,01; ***p<0,001) or Student´s t-test (#p<0,05; ##p<0,01; ns, not significant).

Inflammasomes, such as NLRP3, require the adaptor molecule ASC to activate caspase-1 (53). Upon activation, ASC assembles into large protein complexes, visualized as “specks” (54). Representative confocal images show GFP-labeled ASC specks (white arrowheads, **Figure 5A**). The number of specks in LPS+NP-treated THP-1 ASC-GFP cells was significantly higher than in cells exposed to medium or LPS alone and comparable to the positive control (LPS+DNA, p ≤ 0.05) (**Figure 5B**), indicating that NPs triggered ASC complex assembly. Flow cytometry confirmed a higher frequency of ASC^+^ cells following LPS+NP or LPS+DNA treatment compared with resting cells (**Figures 5C, D**), with NP-induced ASC^+^ cell ratios similar to the positive control.

Consistent with inflammasome activation, hIL-1β secretion was elevated in LPS+NP-and LPS+DNA-treated cells compared with medium or LPS alone (p<0.05) (**Figure 5E**). Pharmacological inhibition of phagocytosis (cytochalasin D), cathepsin B (CA-074Me), or caspases (Z-VAD-FMK) significantly suppressed LPS+NP-induced mIL-1β and mIL-18 secretion (p<0.001) (**Figure 5F**), indicating that NP-mediated inflammasome activation depends on cathepsin B and caspase-1.

**Figure 6:**
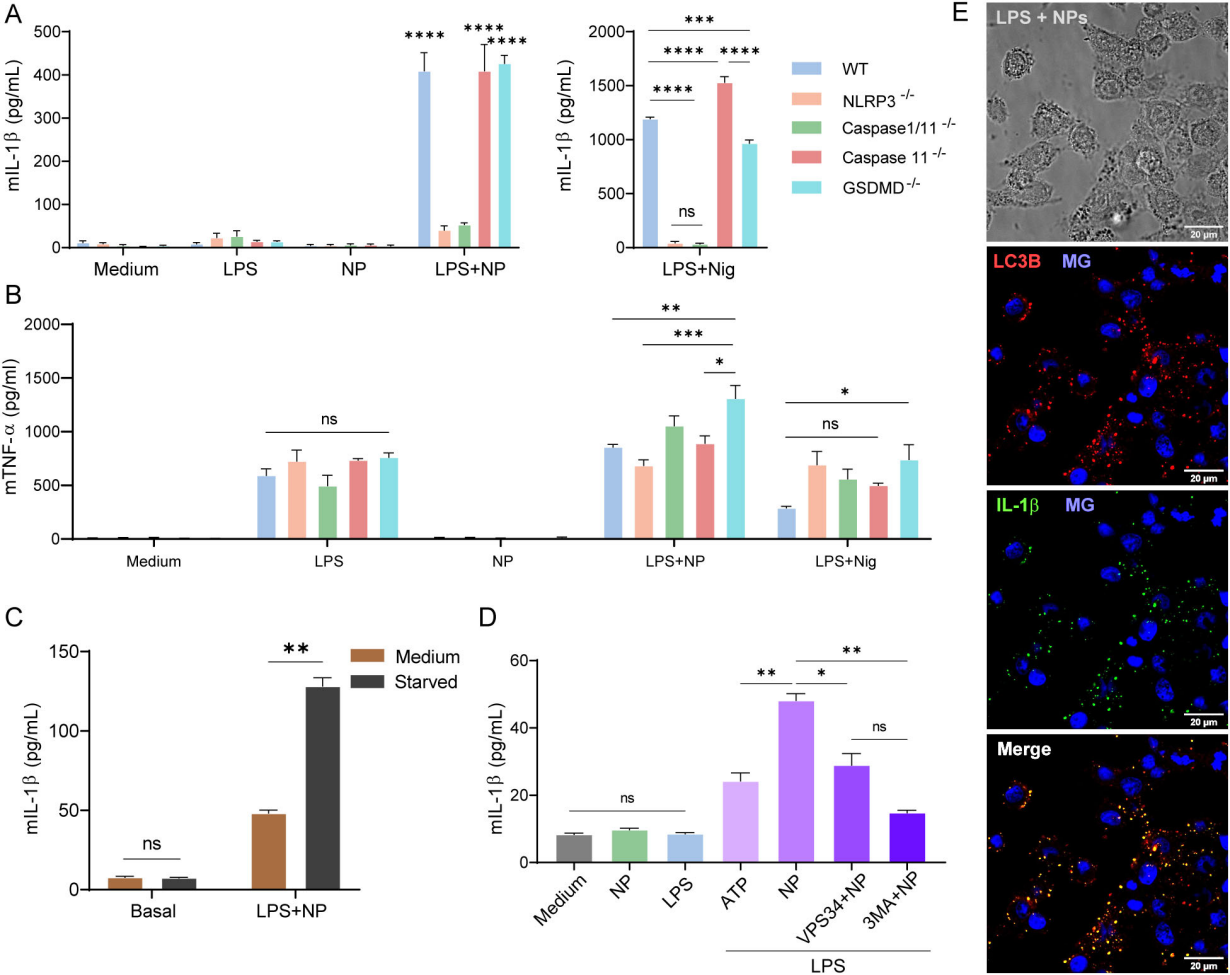
Inflammasome pathway analysis in BMDMs and J774 macrophages and autophagy-dependent regulation of IL-1β secretion. Bone-marrow derived macrophages (BMDMs) from C57BL/6 (WT), NLRP3^−/−^, Casp1/11^−/−^, Casp11^−/−^, GSDMD^-/-^ mice were stimulated with LPS, NP, or LPS+NP, and supernatants were analyzed for **(A)** mIL-1β, **(B)** mTNFα by ELISA. To assess the role of autophagy, J774 macrophages were primed with LPS (3 h) and then exposed to NP. After 6 h, cells were either maintained in medium or subjected to starvation by incubation with Earle’s Balanced Salt Solution (EBSS) for an additional hour. Supernatants were collected, and **(C)** mIL-1β was quantified by ELISA. J774 macrophages were also stimulated for 6 h with NP in the presence of the autophagy inhibitors VPS34-IN1 or 3-methyladenine (3-MA), and **(D)** mIL-1β levels were quantified. **(E)** Confocal microscopy showing co-localization of mIL-1β with the autophagosome marker LC3B after 6 h of stimulation with LPS+NP (MG: Methyl green). Data are expressed as mean ± SEM. Statistical significance was determined by One-way ANOVA: *P<0,05; **P<0,01; ***P<0,001; ****p<0,0001; ns, not significant.

To further delineate the mechanism, bone-marrow-derived macrophages from wild-type and inflammasome-null mice (NLRP3^-^/^-^, caspase-1/11^-^/^-^, caspase-11^-^/^-^ and gasdermin D^-^/^-^). were incubated with LPS (1 μg/mL) and NPs (0.5 mM). mIL-1β secretion was absent in cells treated with LPS or NP alone in both wild-type and knockout BMDCs. LPS+NP induced IL-1β production in wild-type, caspase-11^-^/^-^, and GSMD^-^/^-^ BMDCs, whereas NLRP3^-^/^-^ and caspase-1/11^-^/^-^ cells exhibited significantly reduced secretion (p<0.001) (**Figure 6A**). LPS+Nigericin, used as a positive control, triggered IL-1β release in wild-type and caspase-1^-^/^-^ BMDCs, but was largely abrogated in NLRP3^-^/^-^, caspase-1^-^/^-^, and GSDMD^-^/^-^ cells.

In contrast, mTNF-α secretion was comparable across wild-type and knockout BMDCs upon exposure to LPS, LPS+NP, or LPS+Nigericin, with no TNF-α detected in NP-only treatments (**Figure 6B**). LDH measured in the supernatants showed that cell viability was unaffected by any stimuli (**Supplementary Figure S3A**). Analysis of cell lysates revealed minimal differences in active caspase-1 (20 kDa) levels between wild-type and NLRP3^-/-^ cells exposed to LPS+NP, while inactive caspase-1 was detected in resting and LPS+NP-treated cells and absent in caspase-1/11^-^/^-^ BMDCs (**Supplementary Figure S3B**).

As mentioned before, the release of mIL-1β was independent of GSDMD (**Figure 6A**). This prompted us to investigate the mechanism of IL-1β secretion. Among the multiple pathways described in myeloid cells, we focused on autophagy. Since nutrient starvation is known to induce autophagy (55, 56), we stimulated LPS+NP-treated macrophages with EBSS medium. Starvation significantly increased mIL-1β secretion compared with medium (**Figure 6C**). To validate this finding, we used the autophagy inhibitors 3-methyladenine (3-MA), which blocks the early autophagosome formation, and VPS34-IN1, an inhibitor of class III PI3K. Both treatments significantly reduced mIL-1β secretion in LPS+NP-stimulated macrophages (**Figure 6D**). To confirm if autophagy is involved in the secretion of mIL-1β, we measured, as a control, mIL-6, a cytokine released via the canonical ER-Golgi and independent of autophagy. We observed unchanged high levels of mIL-6 in LPS+NP-treated cells, irrespective of EBSS or inhibitor treatment (**Supplementary Figures S3C, D**). Notably, the reduction in mIL-6 with 3-MA was minimal compared to the strong inhibition observed for mIL-1β (**Figure 6D**). Additionally, cell viability was unaffected, as LDH release remained stable across conditions (**Supplementary Figure S3E**). Consistent results were obtained in BMDCs, in which autophagy inhibition also suppressed IL-1β (**Supplementary Figure S3F**). Finally, confocal microscopy revealed co-localization of mIL-1β with LC3B, a central autophagy protein, in LPS+NP-stimulated macrophages (**Figure 6E**), and not in cells incubated with medium, NP or LPS (**Supplementary Figure S3G**).

Overall, these findings confirmed that the NLRP3-dependent inflammasome activation mediates IL-1β production, while its secretion is produced via the autophagy pathway in LPS+NP-treated cells. However, the reduced but not absent levels of mIL-1β in NLRP3^-/-^ cells, together with the detection of active caspase-1 in NLRP3^-/-^ cell lysates (**Supplementary Figure S3B**), suggest that an additional, NLRP3-independent inflammasome pathway may also contribute to mIL-1β production. Besides, the consistent level of soluble LDH in cells exposed to the medium, NP, or LPS+NP suggests that NP-induced activation did not compromise cell viability.

### 3.4 The systemic administration of NP-OVA induces antigen-specific humoral and cellular immune responses

To evaluate the adjuvant potential of PAH/TPP NPs *in vivo*, we first excluded LPS contamination. Using human TLR4 HEK reporter cells, we confirmed that PAH, TPP, and NP induced SEAP activity comparable to non-stimulated controls and lower than 0.01 ng/mL of LPS (**Supplementary Figure S4**).

Next, we analyzed the protein-encapsulation capacity of PAH/TPP NPs. Considering previous findings that these nanoparticles efficiently encapsulate diverse proteins such as lysozyme, cytochrome C oxidase, bovine serum albumin, and glucose oxidase (45), we used ovalbumin (OVA) as a model antigen. The protein loading was carried out during the ionic crosslinking step in the synthesis of the NPs, with OVA incorporated prior to TPP addition, forming PAH/OVA adducts subsequently crosslinked by the TPP anion (**Supplementary Figure S5A**). According to recent results, the protein acts as an additional ionic crosslinker, generating a three-component complex coacervate in which both the protein and TPP interact with PAH (57). In the case of OVA, whose isoelectric point is below physiological pH, the protein exposes a net negative surface charge, favoring interaction with the positively charged PAH chains. Analysis of the supernatant after centrifugation of PAH/OVA/TPP NPs dispersions (NP-OVA) revealed an encapsulation efficiency of approximately 35% of protein encapsulated for 100 µg/mL OVA (**Supplementary Figure S5B**).

**Figure 7:**
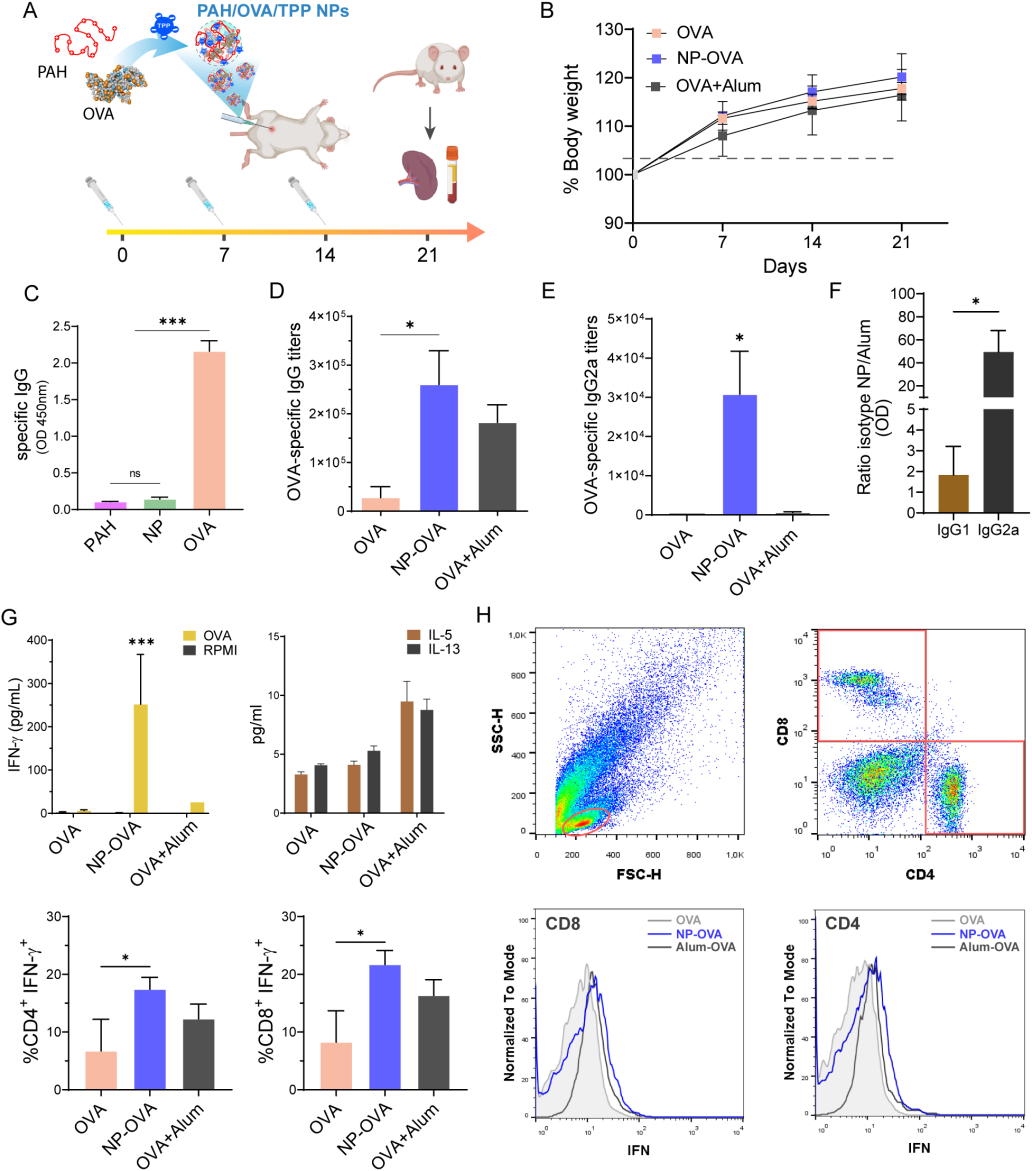
Intraperitoneal immunization of BALB/c mice with NP-OVA and specific humoral and cellular immune responses. **(A)** Immunization scheme; **(B)** Body weight monitoring during the immunization protocol; **(C)** Quantification of serum OVA-specific IgG to PAH, NP and OVA and **(D)** OVA-Specific IgG and **(E)** IgG2a titers in serum and **(F)** ratio of OVA-specific IgG1/IgG2a optical density between NP-OVA- and OVA+Alum-immunized mice by ELISA; **(G)** Quantification of mIFN-γ, mIL-5 and mIL-13 in the supernatants of spleen cells from immunized mice by ELISA (top panel). Frequency of CD4^+^IFN-γ^+^ and CD8^+^IFN-γ^+^ T cells (lower panel). **(H)** Flow cytometry analysis of spleen cells after 4 h incubation with brefeldin A, stained with anti-CD4 (PerCP-Cy5.5) or anti-CD8 (APC); representative gating strategy and Mean Fluorescent Intensity (MFI) are shown. The immunization experiment was performed in duplicate, and all assays were conducted in triplicate. Data are expressed as mean ± SEM. Statistical significance was determined by one-way ANOVA (*p<0,05; ***p<0,001) or Student´s t-test (#p<0,05). ns, not significant.

To analyze the immune response, mice were immunized intraperitoneally with NP-OVA in 3 weekly doses (**Figure 7A**). As a control, mice received OVA formulated with aluminum hydroxide (Alum) (58), an adjuvant approved for human use. No weight loss was observed in any group throughout the experiment (**Figure 7B**). Remarkably, no antibodies against PAH or the NP carrier were detected in NP-OVA immunized animals (**Figure 7C**). Animals that received NP-OVA produced significantly higher levels of serum OVA-specific IgG than mice injected with OVA (p<0.01), reaching titers similar to those induced by OVA+Alum (**Figure 7D**). Subclass analysis revealed that NP-OVA elicited markedly significantly higher titers of OVA-specific IgG2a than OVA+Alum (**Figure 7E**). Accordingly, the IgG1/IgG2a ratio indicated a preferential induction of IgG2a in NP-OVA-immunized animals (**Figure 7F**). To analyze the T cell-mediated immune responses, spleen cells were restimulated *in vitro* with OVA, and cytokines were quantified in culture supernatants. We found that NP-OVA-immunized animals had significantly higher levels of IFN-γ compared with OVA or OVA+Alum groups (p<0.001). Cells from mice that received OVA or OVA+Alum did not secrete IFN-γ (**Figure 7G**). On the other hand, the Th2 cytokines mIL-5 and mIL-13 were reduced in NP-OVA-treated mice compared with OVA+Alum (**Figure 7G**). To further evaluate the cell source of IFN-γ, splenocytes were analyzed by intracellular cytokine staining following *in vitro* OVA stimulation. We found a significantly increased frequency of CD4^+^IFN-γ^+^ and CD8^+^IFN-γ^+^ lymphocytes in NP+OVA-immunized mice (**Figure 7H**). Overall, these findings support that NP-OVA promoted the induction of a Th1-dependent immune response characterized by high titers of OVA-specific IgG and IgG2a, and robust activation of IFN-γ-producing CD4^+^ and CD8^+^IFN-γ^+^ T cells.

To further assess the immunogenicity of NP-OVA, alternative routes of administration were evaluated (**Supplementary Figures S6A, F**). Both administrations were safe in terms of body weights (**Supplementary Figures S6B, G**). Remarkably, intramuscular delivery of a tenfold lower NP-OVA dose elicited strong systemic responses, with increased levels of OVA-specific IgG in both serum and bronchoalveolar lavage (BAL) (**Supplementary Figures S6C, D**) and increased IFN-γ production (**Supplementary Figure S6E**). Our findings showed that a tenfold lower intramuscular dose of NP-OVA compared with intraperitoneal administration elicited a significant increase in OVA-specific IgG in both serum and BAL, together with enhanced IFN-γ production. Comparable humoral and cellular responses were observed after intranasal administration, confirming that NP-OVA induces systemic and mucosal Th1-type immunity irrespective of the immunization route (**Supplementary Figures S6H–J**).

**Figure 8:**
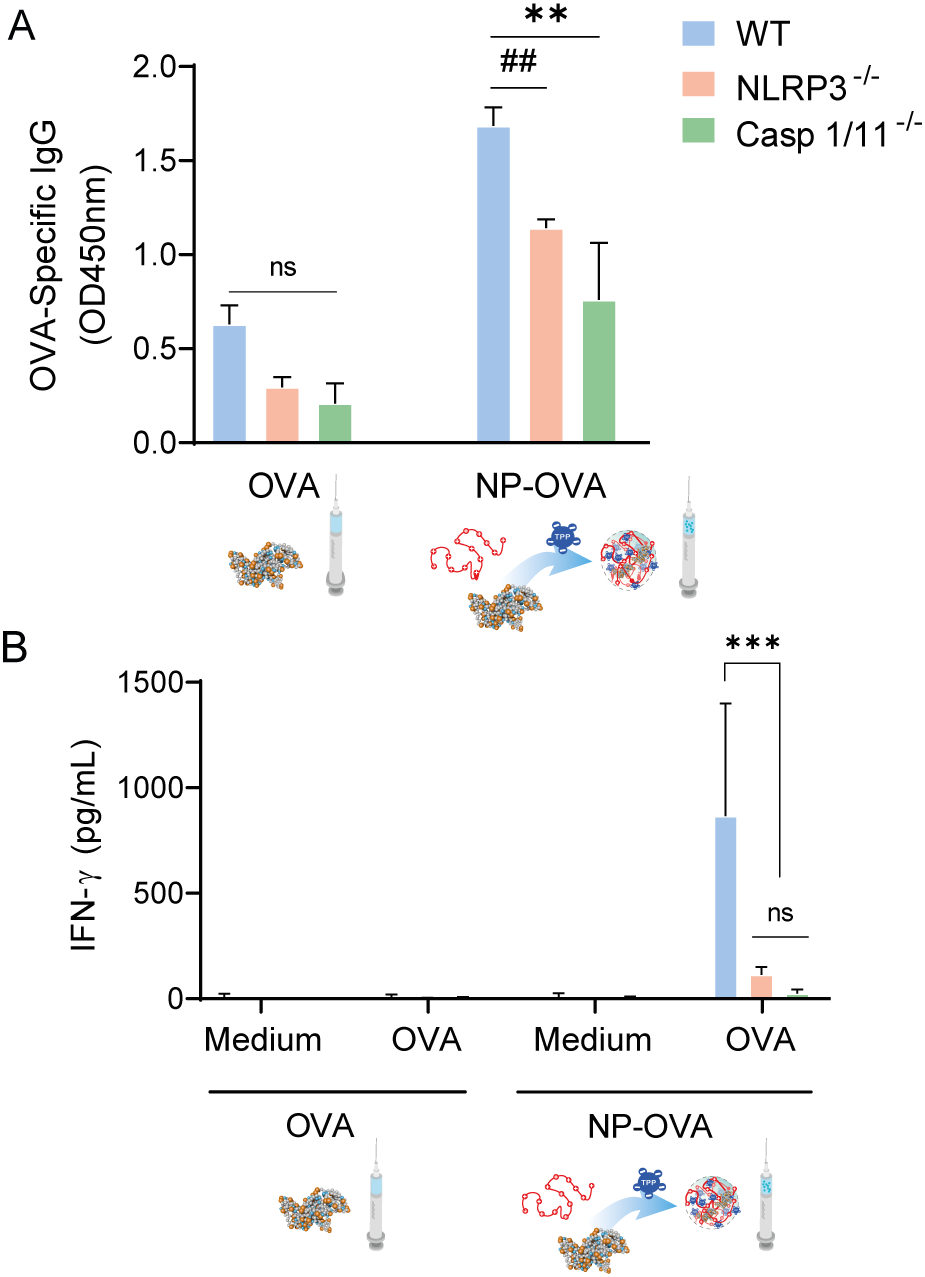
Intraperitoneal immunization with NP-OVA in WT and knockout C57BL/6 mice and analysis of specific humoral and cellular responses. C57BL/6 WT, NLRP3^−/−^, Casp1/11^−/−^ mice were immunized according to the scheme shown in **Figure 6A**. **(A)** Serum OVA-specific IgG (dilution 1:100) measured by ELISA and **(B)** secreted mIFN-γ by spleen cells restimulated *in vitro* with OVA quantified by ELISA. Immunization experiments were performed in duplicate, and all assays were conducted in triplicate. Data are expressed as mean ± SEM. Statistical significance was determined by one-way ANOVA (**p<0,01; ***p<0,001) or Student´s t-test (##p<0,01). ns, not significant.

To investigate the contribution of inflammasome signaling to promote the specific immune response, C57BL/6 mice wild-type (WT) and NLRP3^-/-^ and caspase1/11^-/-^ mice were intraperitoneally immunized with NP-OVA. As shown in **Figures 8A**, both NLRP3^-/-^ and caspase1/11^-/-^ mice exhibited significantly reduced levels of serum OVA-specific IgG compared with WT mice (p<0.01). Analysis of cellular responses revealed that spleen cells from WT mice immunized with NP-OVA secreted higher levels of IFN-γ upon OVA restimulation than WT mice that received OVA alone, as well as with NLRP3^-/-^, caspase1/11^-/-^ immunized with NP-OVA (**Figure 8B**). Together, these findings indicate that NLRP3 and caspase-1 are required for the induction of NP-OVA-mediated Th1 immune responses.

## 4 Discussion

Our findings demonstrate that PAH/TPP NPs are selectively internalized by professional phagocytic antigen-presenting cells, including dendritic cells and macrophages, but not by non-phagocytic epithelial cells. Once internalized, NPs localize within endosomal compartments, induce their disruption, and release their content into the cytosol. This process leads to cellular activation and assembly of the inflammasome, resulting in the secretion of the pro-inflammatory cytokines IL-1β and IL-18. Consistent with previous reports, lysosomal membrane permeabilization represents a central mechanism for particle-mediated inflammasome activation (59, 60). Using specific inhibitors, we confirmed that cathepsin B acts as an early mediator of this pathway (61, 62). Then, using BMDM KO mice, we established that IL-1β secretion induced by NPs is dependent on NLRP3 and caspase-1, but independent of caspase-11 and gasdermin-D, with no evidence of pyroptotic cell death (63). Moreover, we demonstrated that NP-induced IL-1β secretion requires autophagy. This is consistent with previous studies showing that the non-canonical autophagy pathway facilitates the secretion of inflammatory mediators, such as IL-1β and HMGB1 in myeloid cells (64, 65). In macrophages and monocytes, LC3B is essential for the efficient secretion of IL-1β in response to lysosomal damage (56, 66). In this sense, confocal microscopy confirmed IL-1β co-localization with LC3B, supporting a mechanistic link between inflammasome activation and autophagy-mediated IL-1β release. Collectively, these findings show that PAH-based NPs activate phagocytic cells, increase the expression of MHC II and CD86, and trigger IL-1β and IL-18 secretion without compromising cell viability, highlighting their potential as vaccine adjuvants.

Aluminum salts, such as aluminum hydroxide (alum), are among the most widely used adjuvants in licensed vaccines. Their activity relies on depot formation, antigen aggregation to facilitate uptake by APCs, and the activation of the NLRP3 inflammasome to promote adaptive immunity, with low levels of IFN-γ production and low frequency of CD8^+^ T cells and memory (67). *In vivo*, we demonstrated that systemic administration of PAH/TPP NP encapsulating ovalbumin (NP-OVA) elicited robust adaptive immune responses. NP-OVA induced innate immunity through the inflammasome pathway, thereby promoting Th1-driven adaptive humoral and cellular responses. Our findings showed that NP-OVA immunization induced OVA-specific IgG and IgG2a production in serum, along with the expansion of CD4^+^- and CD8^+^-IFN-γ-producing T cells. Th1-dependent isotype synthesis and IFN-γ were confirmed to be NLRP3- and caspase-1-dependent. These findings are consistent with reports that caspase-1–dependent IL-1β and IL-18 secretion promote interferon-γ production and Th1 differentiation (68, 69). Similar to other adjuvants, such as Alum and QS-21, inflammasome priming *in vivo* may be triggered by endogenous danger-associated molecular patterns, such as uric acid, released at the injection site (70, 71).

Additionally, constitutive IL-18 expression in multiple cell types could amplify IL-1β transcription and contribute to the pro-inflammatory cascade during vaccination (72). Remarkably, the *in vivo* induction of OVA-specific CD8^+^T cells indicates that NP-OVA promotes antigen cross-presentation in APCs, likely facilitated by endosomal disruption and antigen release into the cytosol. Further studies are needed to establish whether both macrophages and dendritic cells contribute to this process, which is particularly relevant when considering using PAH-based NP as an adjuvant in an anti-tumoral or viral vaccine. In addition, the intramuscular immunization with a tenfold lower dose of NP-OVA than used intraperitoneally elicited comparable OVA-specific IgG and IFN-γ levels.

While our work primarily focused on systemic immunization, we also demonstrated that intranasal administration of NP-OVA induced comparable Th1-skewed responses, characterized by elevated levels of OVA-specific IgG in serum and bronchoalveolar lavage fluid and enhanced IFN-γ production. This highlights the versatility of PAH/TPP nanoparticles as carriers and adjuvants capable of eliciting both systemic and mucosal immunity.

Our results differ from those of Muñoz-Wolf et al. (73), in the inflammasome pathway involved. While they reported that polymeric nanoparticles of poly-lactic-co-glycolic acid with a diameter of 50–60 nm triggered robust cellular immunity through non-canonical caspase-11- and the gasdermin D-dependent inflammasome activation, we found that PAH-based nanoparticles activated both murine and human APCs via the canonical NLRP3-ASC-caspase-1 axis, inducing IL-1β and IL-18 secretion without pyroptotic cell death (74, 75), and promoted antigen-cross-presentation to trigger CD8^+^ T cells. This mechanistic distinction is significant because Muñoz-Wolf et al. described a division of labor for IL-1β and IL-18 to induce Th1 and CD8+ T cell activation, respectively, which was associated with pyroptotic cell death. In contrast, PAH-based nanoparticles promoted antigen cross-presentation and CD8+ T cell activation through the canonical pathway while preserving cell viability, demonstrating that robust cellular immunity can be achieved independently of pyroptosis.

## Conclusion

In summary, we characterized a novel polyallylamine-based self-assembled nanoparticle as a safe, biocompatible, and effective adjuvant capable of efficiently delivering the encapsulated antigen to phagocytic antigen-presenting cells. NP-OVA immunization induced robust Th1-skewed humoral and cellular immune responses, including OVA-specific IgG2a antibodies and IFN-γ-producing CD4^+^ and CD8^+^ T cells. Mechanistically, PAH-based nanoparticles activated the canonical NLRP3-caspase-1 inflammasome and required autophagy for IL-1β secretion, while avoiding pyroptotic cell death.

Study limitations include the lack of biodistribution analysis and long-term safety and toxicity studies beyond the acute observation period. Additionally, the capacity to induce long-lasting immunological memory and protection against challenge in relevant disease models remains to be established. Despite these limitations, these findings advance our understanding of nanoparticle-based adjuvants and underscore their potential for vaccine development against infectious and non-infectious diseases. Preliminary observations further suggest that PAH/TPP NPs can elicit mucosal as well as systemic immunity, promoting Th1-skewed immune responses, broadening their applicability to diverse vaccination strategies. By integrating innate immune activation with robust adaptive response, this nanosystem represents a promising platform for next-generation vaccines.

## Data Availability

The raw data supporting the conclusions of this article will be made available by the authors, without undue reservation.
