# Supplementary material for: Poly(allylamine)/tripolyphosphate nanocomplex coacervate as a NLRP3-dependent systemic and mucosal adjuvant for vaccines
Source: Front Immunol. 2026 Apr 17;17:1751634. doi: 10.3389/fimmu.2026.1751634 (PMC13133030; doi:10.3389/fimmu.2026.1751634)
Supplement: Supplementary file 1 [file DataSheet1.docx]

Supporting information for:

**Poly(allylamine)/tripolyphosphate Nanocomplex Coacervate as a NLRP3-dependent Systemic and Mucosal Adjuvant for Vaccines**

Gastón P. Rizzo,^1^ Rodrigo C. Sanches,^2^ Camila Chavero,^1^ Daiana S. Bianchi,^1^ Eugenia Apuzzo,^3^ Santiago E. Herrera,^4^ Maximiliano L. Agazzi,^5^ M. Lorena Cortez,^3^ Waldemar A. Marmisollé,^3^ Irene A. Keitelman,^6^ Analía S. Trevani,^6^ Sergio C. Oliveira,^2^ Omar Azzaroni,^3^ Paola L. Smaldini*,^1†^ Guillermo H. Docena.^1†^

^1^Instituto de Estudios Inmunológicos y Fisiopatológicos (IIFP), UNLP, CONICET, asociado a CIC PBA, Facultad de Ciencias Exactas, Departamento de Ciencias Biológicas, La Plata, Argentina (IIFP-UNLP-CONICET).

^2^Department of Biochemistry and Immunology, Institute of Biological Sciences, Federal University of Minas Gerais (ICB-UFMG).

^3^Instituto de Investigaciones Fisicoquímicas Teóricas y Aplicadas (INIFTA), (UNLP, CONICET), 1900 La Plata, Buenos Aires, Argentina.

^4^Instituto de Química de los Materiales, Ambiente y Energía (INQUIMAE), UBA, CONICET, Facultad de Ciencias Exactas y Naturales, Departamento de Química Inorgánica Analítica y Química Física. Buenos Aires, Argentina.

^5^Instituto para el Desarrollo Agroindustrial y de la Salud (IDAS), (UNRC, CONICET), Ruta Nacional 36 KM 601, 5800 Río Cuarto, Argentina.

^6^Laboratorio de Inmunidad Innata, Instituto de Medicina Experimental (IMEX), CONICET, Academia Nacional de Medicina, Buenos Aires, Argentina.

*^7^Department of Immunology, Institute of Biomedical Sciences, University of São Paulo (USP)*

**
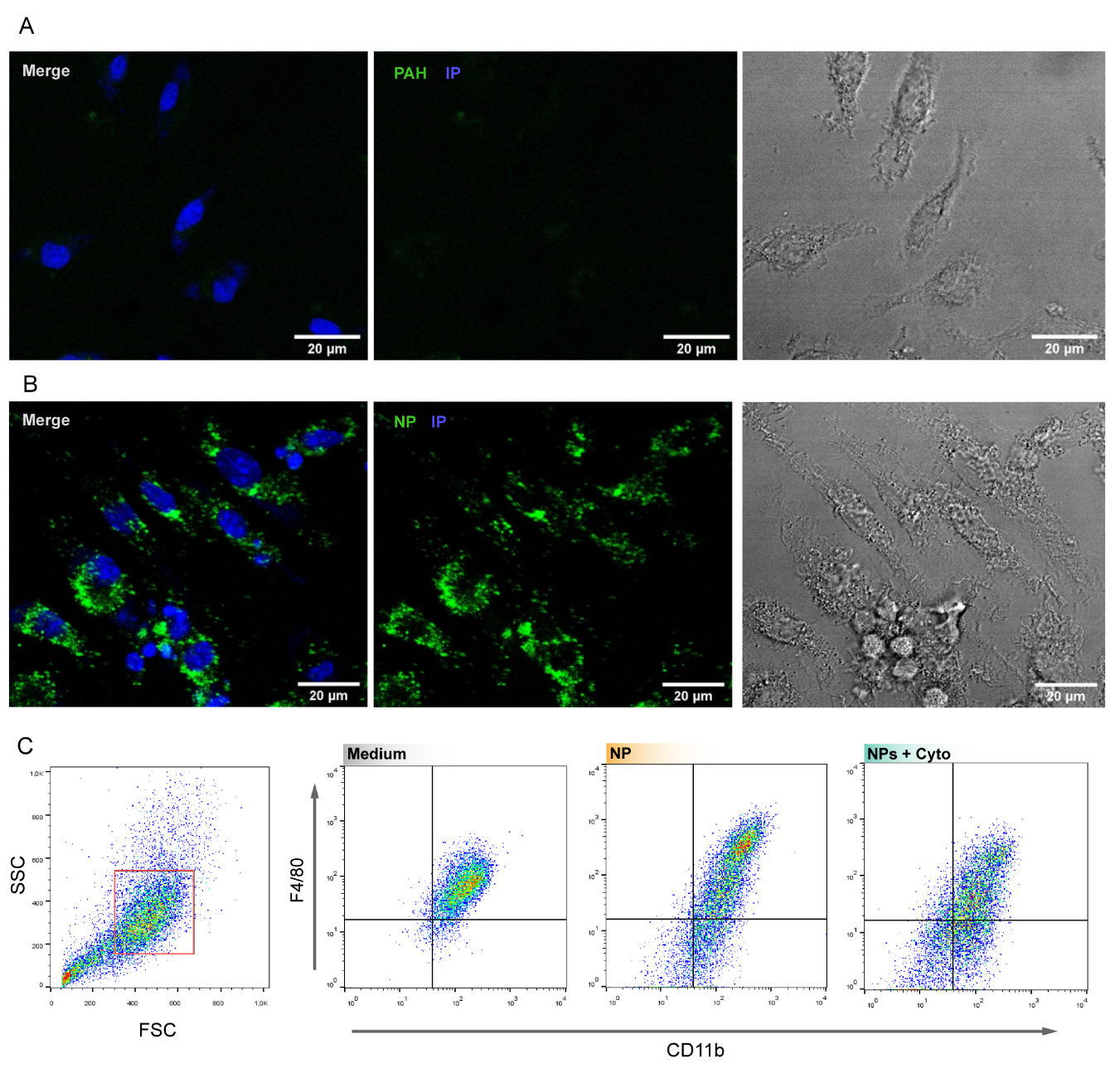
**

**Figure S1: Internalization of (A) PAH-FITC (B) and NP-FITC** in J774 macrophages, visualized by confocal microscopy. Representative images are shown. **(C)** Gating strategy used to select F4/80 CD11b+ macrophages.


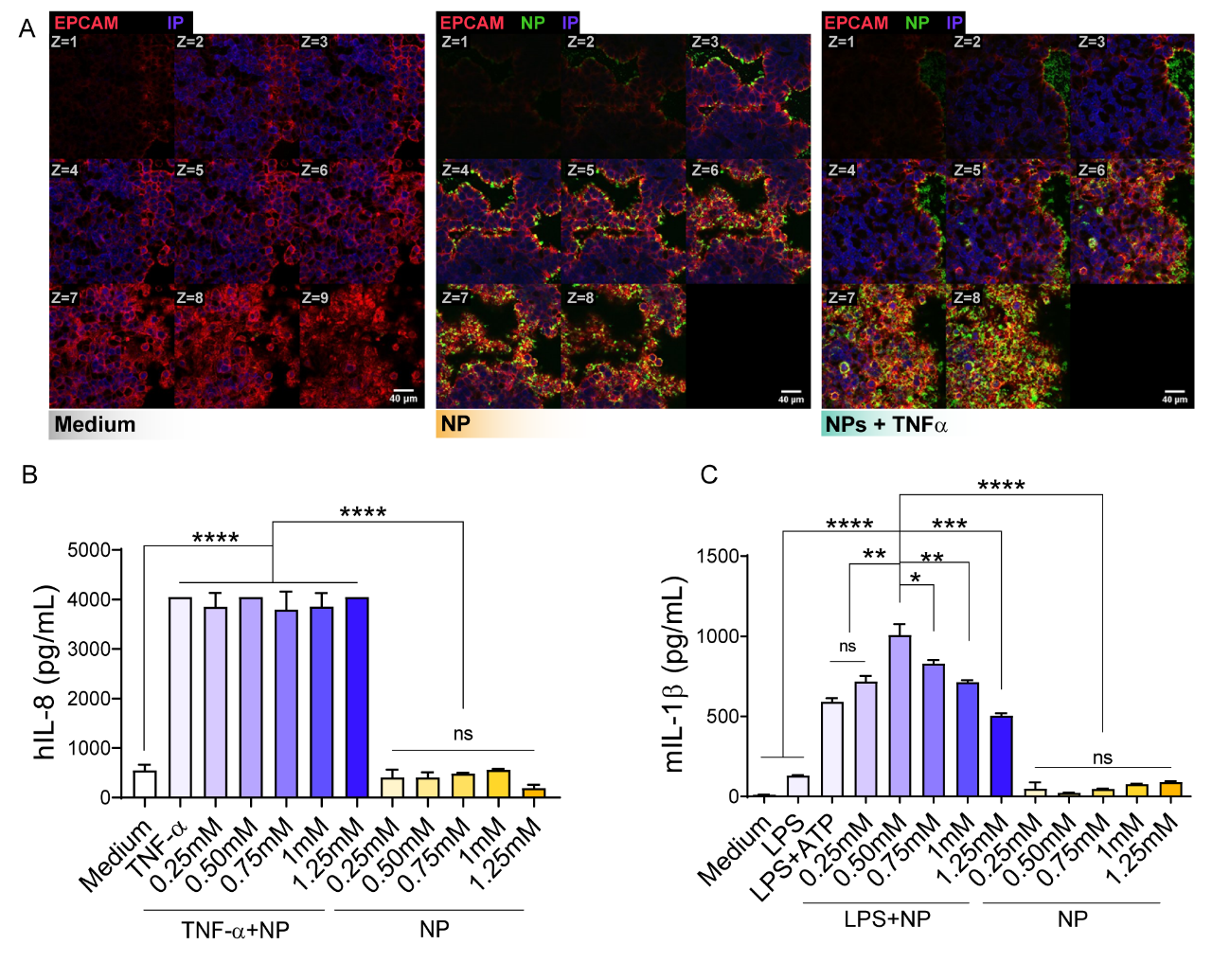


**Figure S2**: **Interaction of NP-FITC with human epithelial cells and cytokine responses.** **(A)** HT-29 cells were scanned at different *z-axis* positions by confocal microscopy; EPCAM was used as an epithelial marker. Representative images are shown. **(B)** Quantification of hIL-8 secretion in HT-29 cells stimulated with medium, TNF-α, TNF-α+NP, or NP alone; **(C)** Quantification of mIL-1β secretion in J774 macrophages exposed to medium, LPS, LPS+ATP, LPS+NP, or NP alone. All experiments were performed in triplicate. Data are expressed as mean ± SEM. Statistical significance was determined by one-way ANOVA (*p<0,05; **p<0,01; ***p<0,001; ****p<0,0001.


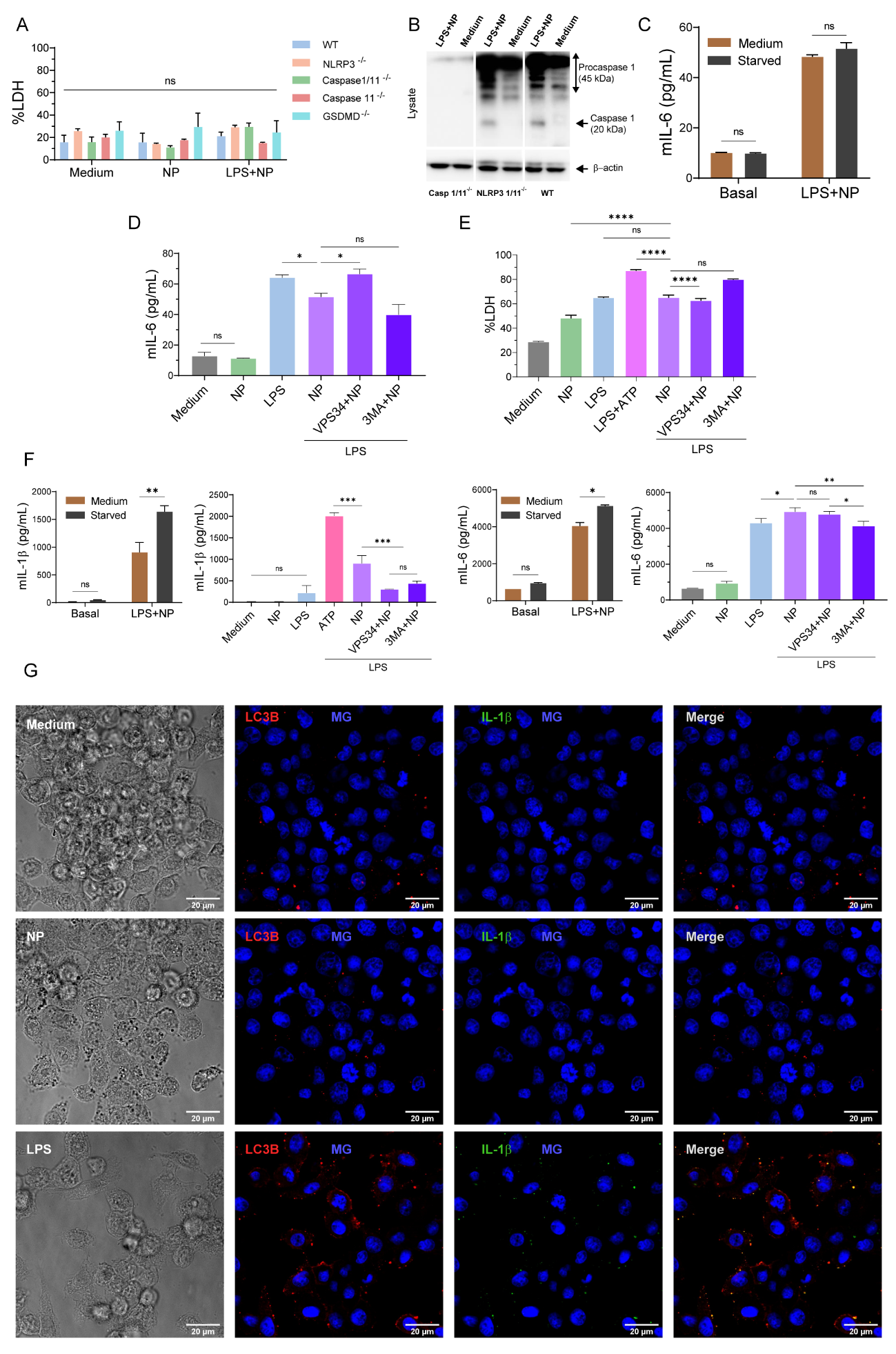


**Figure S3**: **Analysis of** **inflammasome activation in BMDMs from C57BL/6 WT, NLRP3^−/−^, Casp1/11^−/−^, Casp11^−/−^, GSDMD^-/-^ mice, J774 and BMDCs.** BMDMs were stimulated with medium, NP, or LPS+NP. **(A)** LDH release was quantified in the supernatants and **(B)** caspase-1 activation was analyzed by immunoblotting in cell lysates using an antibody specific for the p20 subunit (20kDa) or the procaspase-1 (45kDa). J774 cells were left unstimulated or stimulated with LPS, followed 3 h later by NP treatment for 6 h, in the presence or absence of autophagy inhibitors VPS34-IN1, 3-methyladenine (3-MA), and Bafilomycin A1 (Baf A1). Cells were then maintained in medium or transferred to EBSS for an additional hour of culture. Supernatants were analyzed for **(C-D)** mIL-6 and **(E)** LDH. **(F)** Quantification of mIL-1β and mIL-6 in the supernatants of BMDC after treatment with autophagy inhibitors. **(G)** Confocal microscopy showing mIL-1β-LC3B colocalization in J774 macrophages (MG: Methyl green). Data are expressed as mean ± SEM. Statistical significance was determined by One-way ANOVA: *P<0,05; **P<0,01; ***P<0,001; ****p<0,0001.

**
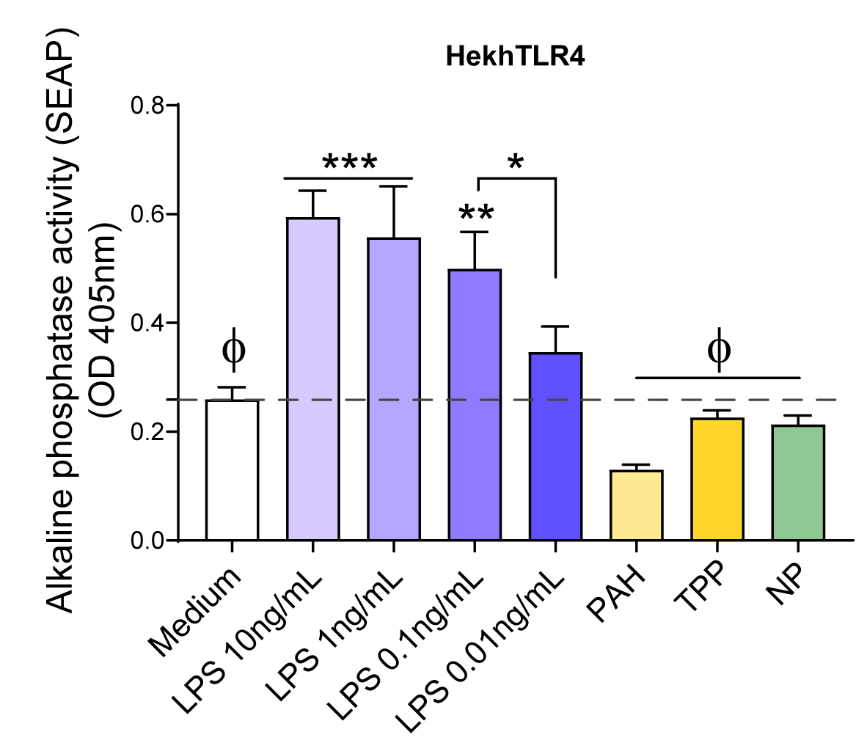
**

**Figure S4**: **Analysis of the stimulation of the HEK-hTLR4 cell line with LPS**. Different concentrations of LPS were included as positive controls; and SEAP activity was quantified in supernatants. Experiments were performed in triplicate, and data are expressed as mean ± SEM. Statistical significance was determined by one-way ANOVA (*p<0,05; **p<0,01; ***p<0,001; Φ, significantly different from LPS).


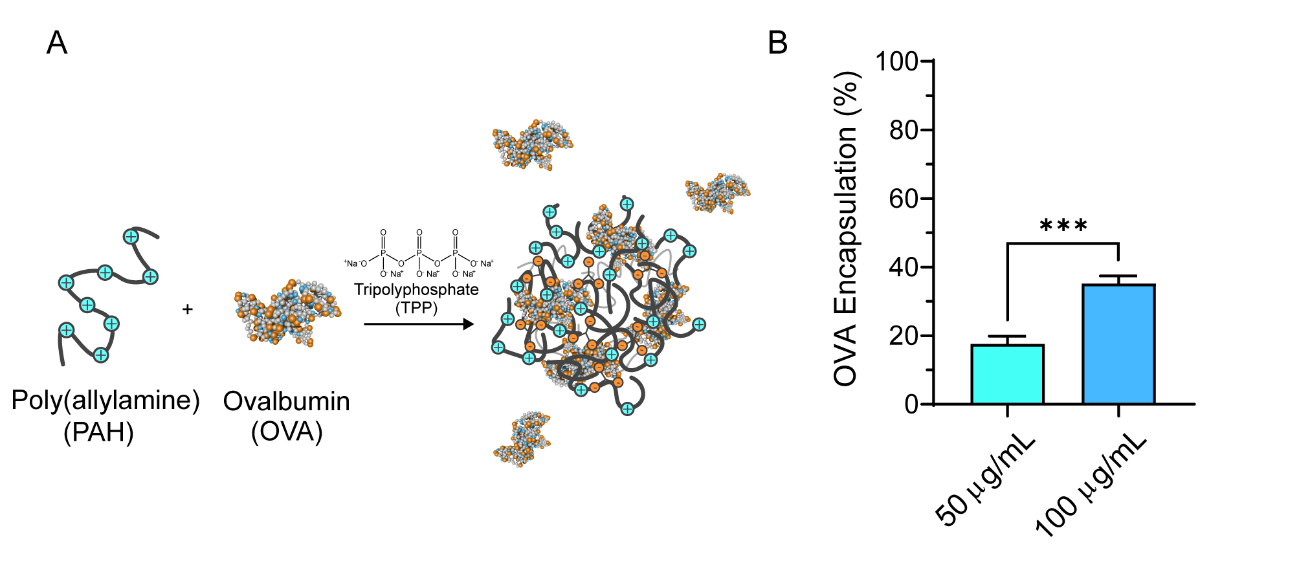


**Figure S5: Analysis of OVA encapsulation by nanoparticles. (A)** Scheme of protein uptake during PAH/TPP NP formation. Because OVA carries a net negative charge at the working pH, free proteins act similarly to PAHs, just like TPP anions. **(B)** Percentage of OVA loading. Free OVA was quantified in the supernatant of the PAH/OVA/TPP NP formulations using the BCA assay. Experiments were performed in triplicate. Data are expressed as mean ± SEM. Statistical significance was determined by Student’s t-test: *** p<0,001.


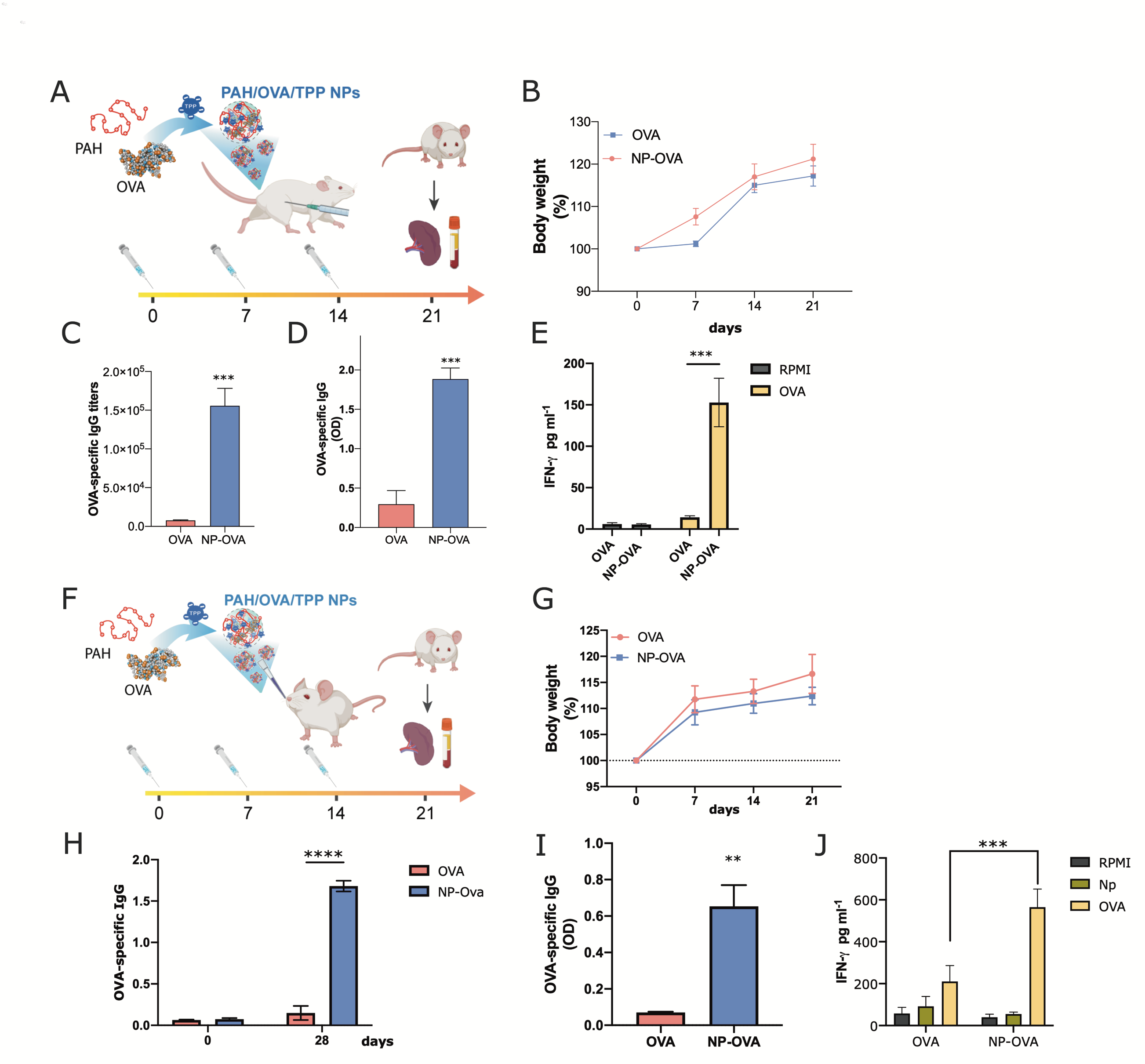


**Figure S6: Immune responses induced by NP-OVA administered via intramuscular or intranasal routes.** (A, F) Immunization schedules; (B, G) Body weight changes; (C, H) Serum OVA-specific IgG levels; (D, I) BAL OVA-specific IgG levels; (E, J) IFN-γ production by splenocytes after *in vitro* OVA stimulation. Immunization experiments were performed in duplicate, and all assays were conducted in triplicate. Data are expressed as mean ± SEM. Statistical significance was determined using ANOVA or Student’s t-test (**p<0.01; ***p<0.001).
